# Unified theory of alpha, mu, and tau rhythms via eigenmodes of brain activity

**DOI:** 10.3389/fncom.2024.1335130

**Published:** 2024-08-26

**Authors:** Rawan Khalil El Zghir, Natasha C. Gabay, P. A. Robinson

**Affiliations:** ^1^School of Physics, University of Sydney, Sydney, NSW, Australia; ^2^Center for Integrative Brain Function, University of Sydney, Sydney, NSW, Australia; ^3^Northern Sydney Cancer Center, Royal North Shore Hospital, St Leonards, NSW, Australia

**Keywords:** EEG, eigenmodes, brain resonances, alpha rhythm, mu rhythm, tau rhythm, neural field theory

## Abstract

A compact description of the frequency structure and topography of human alpha-band rhythms is obtained by use of the first four brain activity eigenmodes previously derived from corticothalamic neural field theory. Just two eigenmodes that overlap in frequency are found to reproduce the observed topography of the classical alpha rhythm for subjects with a single, occipitally concentrated alpha peak in their electroencephalograms. Alpha frequency splitting and relative amplitudes of double alpha peaks are explored analytically and numerically within this four-mode framework using eigenfunction expansion and perturbation methods. These effects are found to result primarily from the different eigenvalues and corticothalamic gains corresponding to the eigenmodes. Three modes with two non-overlapping frequencies suffice to reproduce the observed topography for subjects with a double alpha peak, where the appearance of a distinct second alpha peak requires an increase of the corticothalamic gain of higher eigenmodes relative to the first. Conversely, alpha blocking is inferred to be linked to a relatively small attention-dependent reduction of the gain of the relevant eigenmodes, whose effect is enhanced by the near-critical state of the brain and whose sign is consistent with inferences from neural field theory. The topographies and blocking of the mu and tau rhythms within the alpha-band are explained analogously via eigenmodes. Moreover, the observation of three rhythms in the alpha band is due to there being exactly three members of the first family of spatially nonuniform modes. These results thus provide a simple, unified description of alpha band rhythms and enable experimental observations of spectral structure and topography to be linked directly to theory and underlying physiology.

## 1 Introduction

The first measurement of human brain activity by electroencephalographic (EEG) was made by Berger on 6 July 1924; he observed that the classical ~10 Hz alpha rhythm is the most prominent component of the EEG in healthy awake adults (Berger, 1929). However, the mechanisms behind its spectral structure and spatial topography have yet to be fully explained, despite a century having passed.

The classical alpha rhythm is observed across the whole scalp, but usually has its greatest amplitude over posterior regions (Adrian and Matthews, 1934; Niedermeyer and Lopes da Silva, 1999; Shaw, 2003). Berger (1929) and others found that the alpha rhythm is blocked by mental effort and visual stimuli; however, this response habituates, which means that the alpha rhythm returns when the conditions remain constant. Accordingly, Berger argued that the alpha rhythm is correlated with attention via a mechanism in which a sensory stimulus generates a localized increase in oscillations in the corresponding sensory center, which then exerts a generalized inhibitory effect upon the remainder of the cortex (Berger, 1929; Gloor, 1969). In contrast, Adrian and Yamagiwa (1935) argued that the alpha rhythm is not a property of the whole cortex, but arises from certain parts of the occipital lobes. However, Jasper (1936) discovered that an alpha rhythm could be recorded from central regions, independently of occipital areas; ~10 Hz rhythms have since been recorded across the whole scalp (Niedermeyer and Lopes da Silva, 1999; Chiang et al., 2008, 2011).

The International Federation of Societies for Electroencephalography and Clinical Neurophysiology (IFSECN, 1974) has given an official definition of the human alpha rhythm in adults as a rhythm (i.e., a spectral peak) at frequencies of 8–13 Hz that occurs during wakefulness over the posterior regions of the brain, and is concentrated in the occipital regions. It is strongest when the subject is relaxed with eyes closed, and tends to decrease in amplitude when the eyes are open. The alpha rhythm is also suppressed, or blocked, by attention, especially visual or mental effort (Berger, 1929; Nunez et al., 1978; Niedermeyer and Lopes da Silva, 1999; Shaw, 2003). This suppression by stimuli is often termed alpha “desynchronization,” because high-amplitude alpha rhythms involve some synchronization of neuronal activity (Kropotov, 2009).

The alpha rhythm in normal individuals varies in frequency, amplitude, morphology, and topography. In any individual, the amplitude of the alpha rhythm varies over time, but is mostly < 50 μV at the scalp for adults (Berger, 1929; Cobb, 1963; Niedermeyer and Lopes da Silva, 1999; Shaw, 2003). Furthermore, the EEG power spectrum of a small minority of healthy individuals does not show any distinct peak within the alpha band (Davis and Davis, 1936; Golla et al., 1943; Niedermeyer and Lopes da Silva, 1999; Chiang et al., 2008). An alpha rhythm with a frequency of 4 Hz develops in babies at the age of 4 months, it increases to 6 Hz at the age of 12 months, 8 Hz at 3 years, and reaches about 10 Hz at the age of 20, before decreasing slightly in older individuals (Dustman et al., 1999; Stroganova et al., 1999; Chiang et al., 2011). Notably, studies of the variation of the alpha rhythm during adulthood show that alpha frequencies decrease slightly with age in healthy adults (Duffy et al., 1984; Aurlien et al., 2004; Valdés-Hernández et al., 2010; Chiang et al., 2011). Moreover, the amplitudes of posterior alpha rhythms observed in children are significantly higher than those for adults, which may be due to the lesser effects of volume conduction in their thinner skulls (Nunez et al., 2001), because volume conduction tends to attenuate the transmission of neural electric fields as they pass through conductive brain tissue, cerebrospinal fluid, skull, and scalp to EEG electrodes (Nunez and Srinivasan, 2006). Since the alpha frequency varies with age (Smith, 1941; Chiang et al., 2011), it is likely linked to myelination or neural maturation, which is consistent with the above observations and the observation that a decrease in the alpha frequency is an indicator of dementia (Samson-Dollfus et al., 1997). Other studies presented an anticorrelation between the alpha frequency and the size of the head (Nunez et al., 1978), but Valdés-Hernández et al. (2010) presented evidence that they are independent.

Within the alpha frequency range, there are other activities that differ from the predominant occipital alpha rhythm by their topography and reactivity (Deuschl and Eisen, 1999), such as the rolandic (or central) mu rhythm. The mu rhythm was originally named by Gastaut et al. (1952) who observed it in scalp EEG over the motor cortex at around 10 Hz. Furthermore, magnetoencephalography (MEG) recordings have shown that the mu rhythm is strongest in areas near the primary sensorimotor cortex (Tiihonen et al., 1991) in the rolandic area of the cortex near the central sulcus. It was also found that during voluntary body movements the mu rhythm is suppressed, analogously to suppression of alpha by mental activity; again, this links mu to the sensorimotor cortex (Hari and Puce, 2017).

Another rhythm within the alpha band is the tau rhythm, which was discovered after showing that maximal reactivity to an auditory tone was found in the temporal part of the head at EEG or MEG frequencies of 8–10 Hz (Tiihonen et al., 1991; Yokosawa et al., 2020). The tau rhythm was captured in MEG recordings with neural sources inferred to be localized in the supratemporal auditory cortex (Tiihonen et al., 1991; Lehtelä et al., 1997; Weisz et al., 2011). The tau rhythm is best observed during drowsiness, unlike the occipital alpha rhythm. It is unaffected by eye opening or closing, but is suppressed by auditory stimuli, further linking it to auditory cortex (Niedermeyer and Lopes da Silva, 1999; Shaw, 2003; Nunez and Srinivasan, 2006).

Overall, the presence of several rhythms within the alpha band with partially overlapping frequencies and different topographies highlights the importance of characterizing and explaining each rhythm's frequency, topography, and reactivity, where the reactivity can be quantified via its change in power in response to external stimuli or mental activity relating to the relevant sensory modality (Hari and Puce, 2017). Studying the blocking of those rhythms is also important to understand their corresponding mechanisms, because it was observed by Skinner (1984) that during attention for a particular sensory stimulus, there is a “desynchronization” of 10 Hz rhythmic activity over the corresponding sensory cortex, corresponding to a reduction in amplitude and decrease in the sharpness of the spectral peak. Such features are exactly what is seen in resonant phenomena in multiple branches of physics when the damping rate increases.

As noted above, multiple spectral peaks exist in the conventional alpha band, each associated with a particular area of the brain and differently affected by experimental conditions (Samson-Dollfus et al., 1997). It has been found in healthy individuals that the alpha peak can be split into two sub-peaks separated by up to 1–2 Hz (Nunez et al., 1978; Robinson et al., 2003; Nunez and Srinivasan, 2006; Chiang et al., 2008). In a study of the EEGs of 100 healthy adult subjects, Chiang et al. (2008) found that about 48% of them had a single alpha peak; however, another 48% had two distinguishable subpeaks, which often overlapped. This so-called “split-alpha” spectral structure is thus a common feature of the alpha rhythm in healthy individuals (Nunez and Srinivasan, 2006; Chiang et al., 2008, 2011). Chiang et al. (2008) argued that individuals with a single alpha peak may actually have overlapping peaks whose frequencies are too similar to be distinguished. In most individuals, the alpha-peak frequency is higher at the back of the head than the front–typically by 0.5–1 Hz (Klimesch, 1999; Shaw, 2003; Chiang et al., 2008). Chiang et al. (2008) showed examples (in their Figure 7) of the alpha resonance at various scalp electrodes for a set of individuals, who were classified according to whether their spectra displayed: (i) no distinct alpha peak; (ii) a single alpha peak; or (iii) two alpha peaks.

Proposed mechanisms for the generation of alpha peaks have included: (i) Spatially localized cellular generators, or “pacemakers,” each producing activity with a specific frequency. This theory has been strongly criticized for its lack of explanatory power because it is an *ad hoc* description that requires a new pacemaker for every spectral peak and every spatial concentration of activity (Nunez and Srinivasan, 2006). Furthermore, the pacemaker theory can neither explain the concurrent activation and deactivation of peaks during the transition from wake to sleep (Robinson et al., 2001), nor the frequency relationships between alpha and beta peaks, for example (Valdés-Hernández et al., 2010; van Albada and Robinson, 2013). (ii) Cortical or corticothalamic eigenmodes, where the breaking of degeneracy between modes caused by cortical asymmetry can result in the splitting of the alpha peak (Nunez, 1996; Nunez et al., 2001; Robinson et al., 2001, 2003; Nunez and Srinivasan, 2006). (iii) Non-uniformities in the corticothalamic time delay, which is the time taken by a signal to travel a round trip between the cortex and the thalamus provokes the generation of peaks through a resulting resonance in reciprocal interaction between the thalamus and cortex, thereby potentially leading to the splitting of the alpha peak (Robinson et al., 2003) even in the case of continuously changing local delay.

Neural field theory (NFT) averages neural quantities over scales of a few tenths of a millimeter, and various versions have been widely used to interpret and describe key features of medium-to-large scale brain activity, with local dynamics coupled across regions by corticocortical white matter axons (Wilson and Cowan, 1973; Lopes da Silva et al., 1974; Freeman, 1975; Amari, 1977; Nunez et al., 1978, 2001; Jirsa and Haken, 1996; Nunez, 1996; Wright and Liley, 1996; Robinson et al., 1997, 2002; Ermentrout, 1998; Coombes, 2005; Robinson, 2005; Nunez and Srinivasan, 2006; Deco et al., 2008; Pinotsis et al., 2013). Of particular relevance here has been application of NFT to the corticothalamic system, which is found to be responsible for the production of EEG and fMRI signals as well as many other observable linear and nonlinear phenomena of the brain (Robinson et al., 2002, 2016; Robinson, 2005, 2012; Deco et al., 2008). Corticothalamic NFT predicts the existence of eigenmodes of activity on the cortical surface at length scales detectable by EEG or MEG. Those brain eigenmodes and their dynamics can be described in terms of physiological parameters such as corticocortical, intrathalamic, corticothalamic gains, and inverse synaptodendritic decay and rise times (Robinson et al., 1997, 2002, 2016). As for the eigenmodes of a vast variety of physical systems, the eigenmodes of neural activity on the convoluted cortical surface derived from NFT form a complete basis set such that any brain activity or other quantity can be decomposed in terms of a weighted sum of those eigenmodes (Zwillinger, 1997; Robinson et al., 2001, 2016; Pang et al., 2023). Previous work has also shown that eigenmodes of the convoluted cortex are closely related to spherical harmonics by considering cortical folding as a first-order perturbation from spherical geometry (Gabay and Robinson, 2017).

Eigenmodes represent the building blocks of normal brain dynamics and have also yielded fruitful results regarding brain connectivity via spectral analysis (Robinson et al., 2014, 2016, 2021b; Gao and Robinson, 2020; Henderson et al., 2020; Robinson, 2021). For example, recent work employed just two eigenmodes to reproduce the observed spatiotemporal structure of “echo correlations” between visual stimuli and resulting evoked alpha activity (VanRullen and Macdonald, 2012; Robinson et al., 2018). Another recent study estimated the alpha frequencies corresponding to the first nine brain eigenmodes by approximating the transfer function in corticothalamic NFT (Gabay et al., 2018). However, those frequencies were estimated for corticothalamic loop delay (*τ*) without any spatial nonuniformity, whereas a previous study argued that a variation of −10 to +10 ms in the corticothalamic time delay between the back and the front of the head relative to its mean value of ~80 ms could result in a ~1 Hz splitting of the alpha frequency (Robinson et al., 2003).

In the present work we apply eigenmode analysis systematically to explore the frequency range and topography of the classical alpha, mu, and tau rhythms. This framework estimates the alpha frequency of brain activity in each NFT spatial eigenmode, and when these modes are superposed. Because each spatial mode has a different eigenvalue and characteristic wave number *k*, its corresponding frequency peak is shifted from that of the lowest mode by a different amount (we term this the *k*-effect), and we derive an expression for the resulting frequency splitting in terms of physiological parameters of NFT. The present work also incorporates different values of the corticothalamic time delay *τ* for the other eigenmodes (termed the *τ*-effect here), by treating *τ* as having a mean value plus a perturbation whose effects are expressed in terms of the expectation value of *τ* for each mode. This framework is also used to consider the effect of the variation of corticothalamic feedback loop gain (termed the *G*-effect here) on the splitting of the alpha peak and on the variation of the alpha power between the back and the front of the head, where each mode has a corresponding expectation value of loop gain. This paper also explores the topography of the mu and tau rhythms using the same eigenmode approach.

The structure of the paper is as follows, Section 2 provides an overview of the corticothalamic NFT and the modal expansion. Section 3 analyzes the alpha frequency shift and spectral structure due to the *k*-, *τ*-, and *G*-effects. It describes a possible mechanism of alpha blocking and analyzes cortical alpha topography for both single and double alpha peaks. Results for mu and tau rhythms are also provided. Finally, in Section 4 we summarize and discuss the results.

## 2 Materials and methods

In this section, we first summarize the relevant corticothalamic NFT from prior work (Wright and Liley, 1996; Robinson et al., 1997, 2002, 2004; Robinson, 2005; Abeysuriya et al., 2014), explain its main parameters, and outline the derivation of the corticothalamic transfer function. We then analyze the spatiotemporal dynamics of the brain in terms of eigenmodes.

### 2.1 Corticothalamic neural field theory

**Figure 1:**
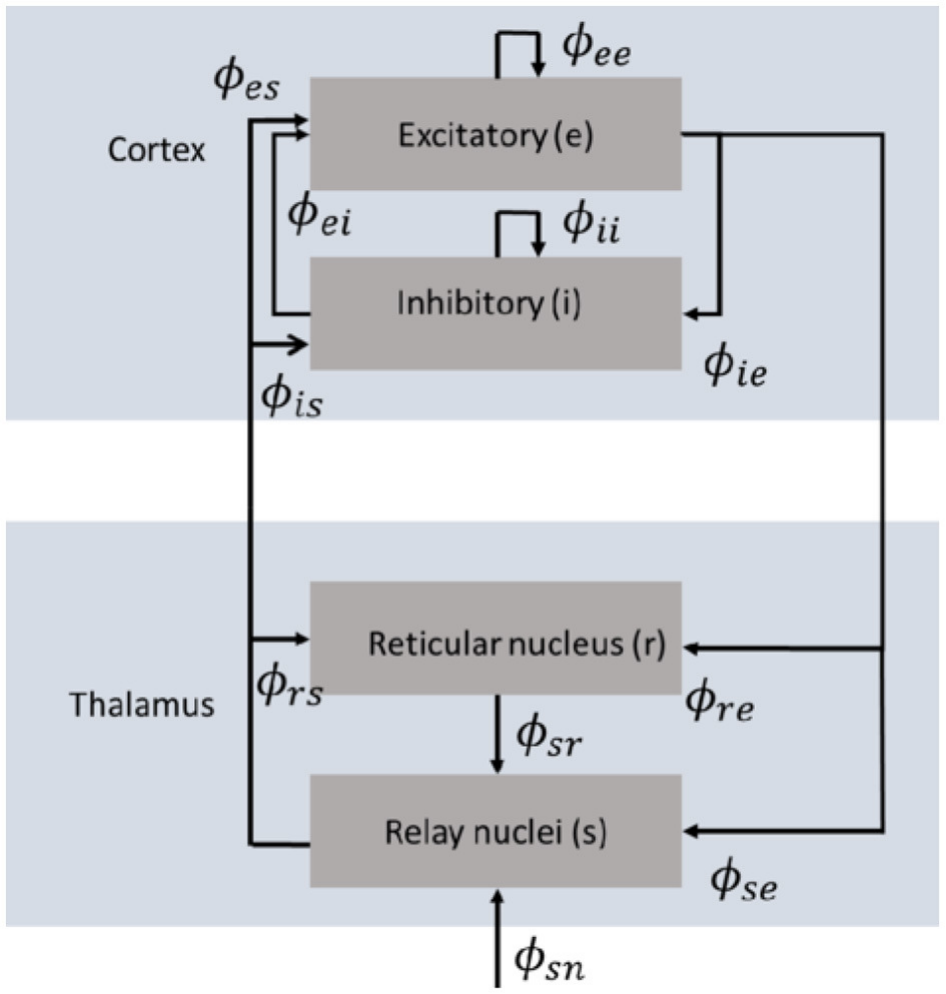
Schematic diagram of the corticothalamic model that incorporates key anatomic connectivities between spatially extended neural populations, where *ϕ*_*ab*_ is the activity reaching population *a* due to signals from population *b* (Gabay and Robinson, 2017).

Neural field theory has been widely used to interpret and reproduce key features of experimental findings in EEG, fMRI, seizure dynamics, coherence and correlations, and evoked response potentials (Robinson et al., 1997, 2002; Rennie et al., 2002; Robinson, 2003; Deco et al., 2008). It averages neural quantities over scales of a few tenths of a millimeter. Here, we focus on the key aspects of NFT relevant to our framework. The neural field model consists of cortical excitatory (*e*) and inhibitory (*i*) populations, thalamic specific relay populations (*s*), thalamic reticular populations (*r*), and external sensory inputs (*n*). This model incorporates key anatomic connectivities between those populations, as shown in Figure 1, where *ϕ*_*ab*_ is the mean activity field reaching population *a* due to signals from population *b*. The strength of connection to population *a* from population *b* is


(1) $$\begin{array}{l} \nu_{ab}=s_{ab}N_{ab}, \end{array}$$

where *s*_*ab*_ is the mean time-integrated strength of the response in neurons *a* per incoming signal from neurons *b*, and *N*_*ab*_ is the mean number of synapses to neurons *a* from *b*. Population *a*'s average firing rate *Q*_*a*_ can be approximated as a nonlinear sigmoid function of its corresponding average membrane potential *V*_*a*_ relative to resting, such that


(2) $$\begin{array}{l} Q_{a}=S\left(V_{a}\right)=\frac{Q_{\max}}{1+\exp\left[-\left(V_{a}-\theta \right)/\sigma^{\prime }\right]}, \end{array}$$

where *Q*_max_ is the maximum firing rate, *θ* is the mean threshold voltage, and $\sigma^{\prime }\pi /\sqrt{3}$ is the standard deviation of the threshold distribution.

Due to synaptodendritic dynamics and soma capacitance, presynaptic inputs to neurons *a* from various types of neurons *b* are summed after being filtered and smeared out in time, giving rise to the potential *V*_*a*_ such that


(3) $$\begin{array}{l} V_{a}\left(\text{r},t\right)=\underset{b}{\sum }V_{ab}\left(\text{r},t\right). \end{array}$$

Inputs drive contributions to the local population response via the following equation:


(4) $$\begin{array}{l} D_{\alpha \beta }\left(t\right)V_{ab}\left(\text{r},t\right)=\nu_{ab}\phi_{ab}\left(\text{r},t-\tau_{ab}\right), \end{array}$$

where *D_αβ_* is the operator


(5) $$\begin{array}{l} D_{\alpha \beta }\left(t\right)=\frac{1}{\alpha \beta }\frac{d^{2}}{dt^{2}}+\left(\frac{1}{\alpha }+\frac{1}{\beta }\right)\frac{d}{dt}+1, \end{array}$$

**r** is the position vector on the 2D cortical sheet, 1/*β* and 1/*α* are the rise and decay times, respectively, of the potential at the cell body elicited by an impulse response at the dendritic synapse, and *τ*_*ab*_ is the time delay due to anatomical separations between neural populations *a* and *b*. The only nonzero time delays correspond to propagation from cortex to thalamus and vice versa, with *τ*_*ab*_ ≈ 0 in the case of intrathalamic and intracortical connections; propagation across the cortex via white matter connections is discussed in the next paragraph.

Propagation between various spatial points **r** across the cortex are due to white matter connections/fibers from excitatory cortical pyramidal neurons (Robinson et al., 2002, 2004; Robinson, 2005). The cortex is approximated as a two-dimensional sheet, while the coordinates in the thalamus are linked one-to-one to those in the cortex via the primary topographic mapping, such that, in the thalamus, the dimensional map coordinate denotes a rescaled physical dimension. The rescaled thalamic coordinate is determined by multiplying the physical coordinate by the ratio of the cortical scale to the thalamic scale (Robinson, 2005).

The following equations show the conversion of the source firing rates *Q*_*b*_(**r**, *t*) into the axonal signal, such that


(6) $$\begin{array}{l} D_{ab}\left(\text{r},t\right)\phi_{ab}\left(\text{r},t\right)=Q_{b}\left(\text{r},t\right), \end{array}$$

(7) $$\begin{array}{l} D_{ab}\left(\text{r},t\right)=\frac{1}{\gamma_{ab}^{2}}\frac{\partial^{2}}{\partial t^{2}}+\frac{2}{\gamma_{ab}}\frac{\partial }{\partial t}+1-r_{ab}^{2}\nabla^{2}, \end{array}$$

where the propagation of a mean activity field *ϕ*_*ab*_(**r**, *t*) obeys a damped wave equation with *γ*_*ab*_ = *v*_*ab*_/*r*_*ab*_ is the temporal damping rate, *r*_*ab*_ is the mean characteristic range of axons to population *a* from population *b*, and *v*_*ab*_ is the propagation velocity in axons to population *a* from population *b*. We can write *r*_*ab*_ ≈ 0 for *b* = *i, r, s*, yielding *D*_*ab*_ = 1 because the mean axonal ranges for all populations except for excitatory cortical neurons are very short. The Laplacian operator in Equation 7 incorporates the dominant, short-range (few cm), approximately isotropic connectivity (Robinson et al., 1997, 2016; Braitenberg and Schüz, 1998; Henderson and Robinson, 2014; Pang et al., 2023), and the effects of cortical curvature; it does not embody inhomogeneities in long-range connectivity, but these have been shown to be perturbations on the dominant contribution (Henderson and Robinson, 2014).

It has been found experimentally that the number of intracortical synapses to different types of cortical neurons is closely proportional to the numbers of neurons involved of each type (Braitenberg and Schüz, 1998), which implies then *N*_*ib*_ = *N*_*eb*_ for all *b* in the present case. If we assume that the mean input stimulus *ϕ*_*sn*_ is not too large, the system has a fixed point with low firing rates (Robinson et al., 1997, 2002). If perturbations around this point are not too large, we can make a linear approximation where we henceforth treat each dynamic quantity (*ϕ*_*ab*_, *Q*_*a*_, *V*_*a*_) as a linear perturbation from its steady state value, which is denoted by the superscript (0). So


(8) $$\begin{array}{l} Q_{a}\left(\text{r},t\right)=\rho_{a}V_{a}\left(\text{r},t\right), \end{array}$$

and


(9) $$\begin{array}{l} \rho_{a}=\frac{dS\left(V_{a}\right)}{dV_{a}}{|}_{V_{a}=V_{a}^{\left(0\right)}}, \end{array}$$

is the derivative with respect to voltage of the sigmoid function, evaluated at the steady state.

Fourier transforming Equations 4–6 yields


(10) $$\begin{array}{l} V_{ab}\left(\text{k},\omega \right)=L\left(\omega \right)\nu_{ab}\phi_{ab}\left(\text{k},\omega \right)e^{i\omega \tau_{ab}}, \end{array}$$

(11) $$\begin{array}{l} L\left(\omega \right)={\left(1-\frac{i\omega }{\alpha }\right)}^{-1}{\left(1-\frac{i\omega }{\beta }\right)}^{-1}, \end{array}$$

(12) $$\begin{array}{l} D_{ab}\left(\text{k},\omega \right)\phi_{ab}\left(\text{k},\omega \right)=Q_{b}\left(\text{k},\omega \right), \end{array}$$

where **k** is the wave vector, *ω* is the angular frequency, and *L*(*ω*) embodies a low-pass filter response function. Activity *Q*_*b*_ generates fields of activity *ϕ*_*ab*_ that propagate to affect populations *a*.

#### 2.1.1 Corticothalamic transfer function

Equations 3, 8, 10, 12 represent a set of linear equations for the *V*_*ab*_, *ϕ*_*ab*_, and *Q*_*ab*_. Equation 8 can be used to eliminate the *V*_*ab*_ in favor of the *Q*_*a*_. Then Equation 12 can be used to eliminate the *Q*_*b*_ in favor of the *ϕ*_*ab*_. This yields a single set of linear equations in the *ϕ*_*ab*_, driven by the source *ϕ*_*sn*_ in the system shown in Figure 1. In the corticothalamic system, observable EEG and MEG signals are primarily generated by the *e* population and are approximately proportional to *ϕ*_*ee*_ at the cortex (Robinson et al., 2001). Solution of the linear equations for the *ϕ*_*ab*_ enables all of these quantities to be eliminated in favor of *ϕ*_*ee*_ via linear algebra [see Abeysuriya et al. (2014), for example, for further details of the derivation]. Hence, we focus on *ϕ*_*ee*_ and express it in terms of the external stimulus *ϕ*_*sn*_ via the transfer function, which is given by


(13) $$\begin{array}{l} T\left(\text{k},\omega \right)=\frac{\phi_{ee}\left(\text{k},\omega \right)}{\phi_{sn}\left(\text{k},\omega \right)}, \end{array}$$

(14) $$\begin{array}{l} \;=\frac{B\left(\omega \right)}{k^{2}r_{ee}^{2}+q^{2}\left(\omega \right)r_{ee}^{2}}, \end{array}$$

where Equation 13 is the definition of *T*(**k**, *ω*)


(15) $$\begin{array}{c} q^{2}\left(\omega \right)r_{ee}^{2}={\left(1-\frac{i\omega }{\gamma_{ee}}\right)}^{2}\\ -\frac{1}{1-G_{ei}L}\left[LG_{ee}+\frac{L^{2}G_{es}\left(G_{se}+LG_{sr}G_{re}\right)\exp\left[i\omega \left(\tau_{es}+\tau_{se}\right)\right]}{1-L^{2}G_{sr}G_{rs}}\right], \end{array}$$

(16) $$\begin{array}{l} B\left(\omega \right)=\frac{L^{2}G_{es}G_{sn}e^{i\omega \tau_{es}}}{\left(1-L^{2}G_{sr}G_{rs}\right)\left(1-G_{ei}L\right)}, \end{array}$$

and *G*_*ab*_ is the gain of responses in population *a* due to signals from population *b* such that *G*_*ab*_ = *ρ*_*a*_*ν*_*ab*_. Table 1 shows the parameters used to evaluate the corticothalamic transfer function numerically.

**Table 1:** Model parameters.

**Symbol**	**Quantity**	**Value**	**Units**
*α*	Synaptodendritic decay rate	50	s^-1
*β*	Synaptodendritic rise rate	200	s^-1
*τ*_*es*_	Thalamocortical axonal delay	0.02	s
*τ*_*se*_	Corticothalamic relay axonal delay	0.06	s
*γ*_*ee*_	Cortical damping rate	116	s^-1
*r*_*ee*_	Excitatory axon range	60	mm
*G*_*ee*_	*ee* gain	2.07	-
−*G*_*ei*_	*ei* gain	4.11	-
*G*_*es*_	*es* gain	0.77	-
*G*_*se*_	*se* gain	7.77	-
−*G*_*sr*_	*sr* gain	3.30	-
*G*_*sn*_	*sn* gain	8.10	-
*G*_*re*_	*re* gain	0.66	-
*G*_*rs*_	*rs* gain	0.20	-

The first six lines show nominal parameters of corticothalamic neural field theory based on previous work (Henderson and Robinson, 2014; Gabay et al., 2018). The remaining lines show nominal gain parameters of the corticothalamic transfer function for the eyes-closed waking state based on previous work (Abeysuriya et al., 2014).

#### 2.1.2 Eigenmodes

If we write $D^{\prime }\left(\omega \right)=q^{2}\left(\omega \right)r_{ee}^{2}$, then in the absence of external stimulus the natural modes of the system obey the dispersion equation given by the zeros of the term $k^{2}r_{ee}^{2}+q^{2}\left(\omega \right)r_{ee}^{2}$ in the denominator in Equation 14. If we spatially transform the term involving *k*, this yields


(17) $$\begin{array}{l} q^{2}\left(\omega \right)\phi_{ee}\left(\text{r},\omega \right)=-\nabla^{2}\phi_{ee}\left(\text{r},\omega \right), \end{array}$$

where ∇^2^ is the Laplace-Beltrami operator. This equation is then in a form that can be solved in the finite system corresponding to a brain hemisphere, with appropriate boundary conditions. We analyze the spatiotemporal dynamics of the brain in terms of discrete modes labeled *η*, with eigenvalues *k*_*η*_ that replace the continuous **k** above.

To solve Equation 17 for the eigenmodes, we introduce the ansatz


(18) $$\begin{array}{l} \phi_{ee}\left(\text{r},t\right)=u_{\eta }\left(\text{r}\right)e^{-i\omega_{\eta }t}, \end{array}$$

where *u*_*η*_(**r**) is a spatial eigenmode on the cortical surface oscillating at an eigenfrequency *ω*_*η*_; *η* labels the mode. Substituting Equation 18 into Equation 17 then yields


$$\begin{array}{l} \frac{\nabla^{2}u_{\eta }\left(\text{r}\right)}{u_{\eta }\left(\text{r}\right)}=q^{2}\left(\omega \right). \end{array}$$

In this equation, the left hand side is independent of *ω*, while the right hand one is independent of **r**; hence, they must both be equal to a common constant, which we write as $-k_{\eta }^{2}$. The left side then yields the Helmholtz equation for *u*_*η*_(**r**):


(19) $$\begin{array}{l} \nabla^{2}u_{\eta }\left(\text{r}\right)=-k_{\eta }^{2}u_{\eta }\left(\text{r}\right), \end{array}$$

where *u*_*η*_(**r**) are the eigenmode solutions of the equation and $k_{\eta }^{2}$ are the corresponding eigenvalues. The right side gives $q^{2}\left(\omega \right)+k_{\eta }^{2}=0$, which is the dispersion relation of the mode, as noted earlier.

**Figure 2:**
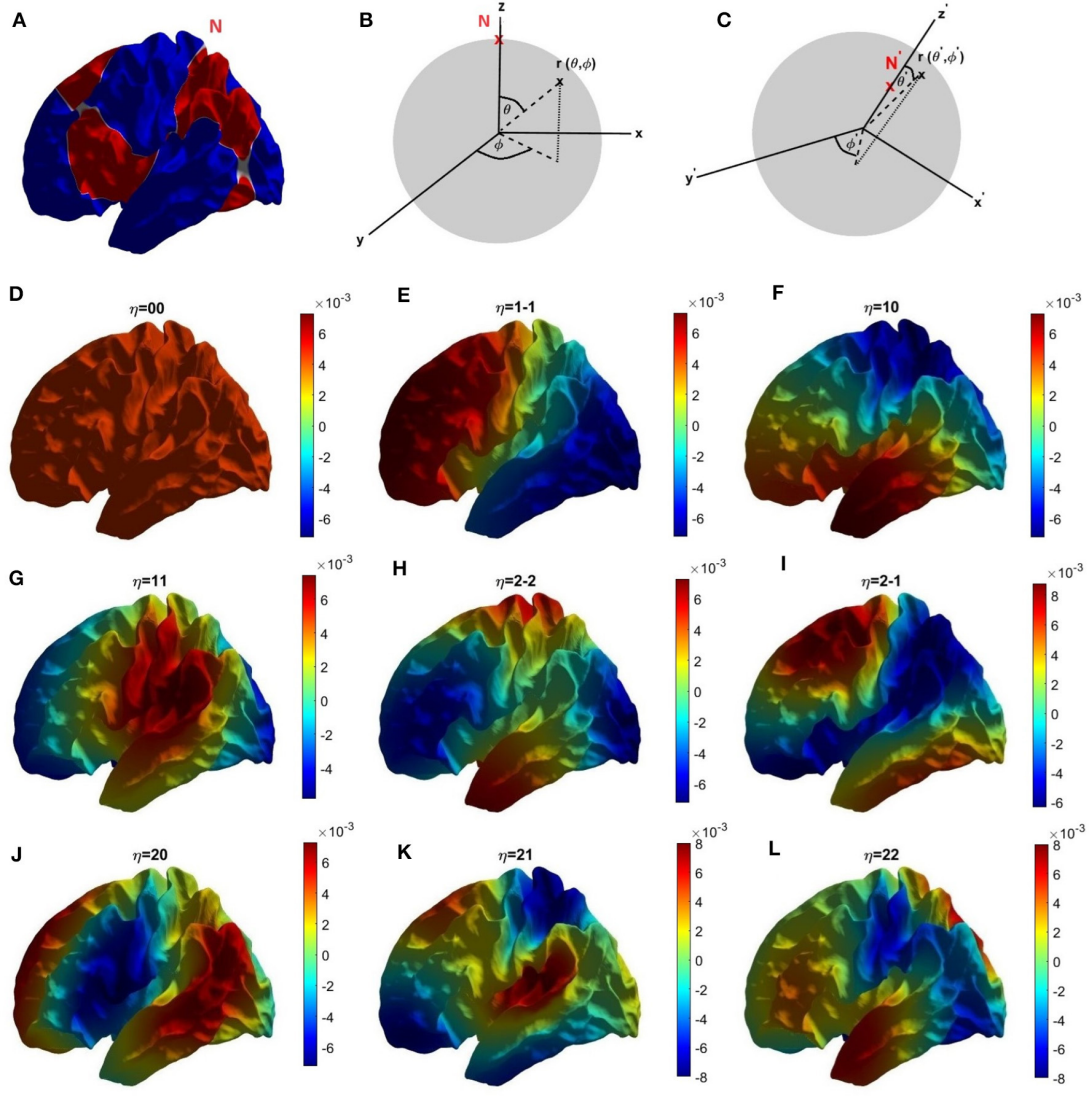
Coordinates and modes. **(A)** Location of the north pole and nodal lines of the first three nonuniform modes on the cortical surface in the left hemisphere. Alternating colors are used to highlight the nodal lines, shown white. **(B)** Parameterization of cortical surface via the Freesurfer mapping to a sphere using the Freesurfer coordinates. **(C)** Our rotated coordinate system, based on the location of our north pole at the intersection of two nodal lines shown in **(A)**. **(D–L)** First nine eigenmodes, labeled with mode number *η* = *lm* with red and blue indicating positive and negative regions, respectively; the color bar is shown at the foot of the figure.

The Helmholtz (Equation 19) is solved numerically on the surface of an average cortical hemisphere, shown in Figure 2A, with this surface provided by the Freesurfer package (Fischl et al., 1999). The Freesurfer software also enables us to establish a coordinate system in which to parameterize these calculations by performing a one-to-one mapping of each point **r** of a cortical hemisphere onto a sphere at a point (*ϑ*, *φ*), where *ϑ* and *φ* are the spherical polar angle and azimuthal angle, respectively (Fischl et al., 1999; Robinson et al., 2016) as shown in Figure 2B. (In essence, the brain hemisphere is computationally inflated into a sphere to establish the mapping). Figure 2C shows the coordinates used in the present work, which have been rotated so as to place their north pole at the intersection of two nodal lines, as for spherical harmonics.

The human cerebral cortex can be approximated as a highly convoluted two-dimensional sheet, a geometry that makes computational analysis and visualization difficult. Consequently, a useful first step is to computationally inflate each cortical hemisphere into a geometric sphere in order to establish a coordinate system while preserving the topology (Fischl et al., 1999). The standard spherical coordinate system on the inflated sphere is then used to label corresponding points on the convoluted surface for the purposes of calculation and visualization. Using this grid of spatial points on the convoluted cortical surface, parameterized by the associated spherical coordinates, the corresponding spatial eigenmodes and their eigenvalues *k*_*η*_ are calculated by solving the Helmholtz (Equation 19) on the convoluted cortex using standard finite-differencing methods (Seo and Chung, 2011; Robinson et al., 2016; Pang et al., 2023). Figures 2D–L show the first nine eigenmodes on the cortex, where we label each mode by writing *η* = *lm* where *Y*_*lm*_ is the spherical harmonic limit of the mode in the limit that the convolutions are removed (Robinson et al., 2016). In this limit, the labels have the values *l* = 0, 1, 2, … and *m* = −*l*, −*l* + 1, …, *l* − 1, *l*. Focusing on just the first four eigenmodes; the first eigenmode (*η* = 00 in Figure 2D) is uniform across the cortex, the second eigenmode (*η* = 1-1, Figure 2E) varies primarily front-to-back, the third eigenmode (*η* = 10, Figure 2F) varies mainly top-to-bottom, and the fourth eigenmode (*η* = 11, Figure 2G) varies chiefly left-to-right.

### 2.2 Modal expansion

In this section we first summarize and apply the modal expansion discussed in Gabay et al. (2018), Babaie-Janvier and Robinson (2018), and El Zghir et al. (2021) to the power spectrum, we then show our predictions for the alpha power spectrum between the front and back of the brain.

The corticothalamic transfer function expressed in Equations 13, 14 represents the cortical response to an input stimulus, and encodes all the information of the linear system. However, that form of the transfer function is transcendental, not easy to work with, and does not link clearly to experimental observations (e.g., resonances in EEG spectrum). Modal-polar expansion simplifies that complicated form of the transfer function by decomposing Equations 13, 14 into two separate parts: the modal part (spatial), and the temporal part as in Equation 18 (El Zghir et al., 2021).

Assuming that the transfer function is spatially symmetric, the set of eigenmodes *u*_*η*_ is complete and orthonormal. Hence, any activity or connectivity can be expressed in terms of those eigenmodes (Robinson et al., 2016; Gao and Robinson, 2020; Robinson, 2021), which means the corticothalamic transfer function in Equation 13 can be expanded in terms of the brain eigenmodes such that


(20) $$\begin{array}{l} T(\text{r},{\text{r}}^{\prime },\omega )=\underset{\eta }{\sum }u_{\eta }(\text{r})u_{\eta }^{*}({\text{r}}^{\prime })T(k_{\eta },\omega ), \end{array}$$

where *u*_*η*_(**r**) are the eigenmodes, and *T*(*k*_*η*_, *ω*) is the temporal part of the transfer function for mode *η*.

#### 2.2.1 Power spectrum

Using the above modal-polar representation of NFT, we can express the spatially dependent power spectrum in terms of the modes, with


(21) $$\begin{array}{l} P\left(\text{r},\omega \right)=\langle |\phi_{ee}\left(\text{r},\omega \right){|}^{2}\rangle , \end{array}$$

(22) $$\begin{array}{l} \;=\langle |\underset{\eta }{\sum }\phi_{ee}\left(k_{\eta },\omega \right)u_{\eta }\left(\text{r}\right){|}^{2}\rangle , \end{array}$$

(23) $$\begin{array}{l} \;=\langle |\underset{\eta }{\sum }T\left(k_{\eta },\omega \right)\phi_{sn}\left(k_{\eta },\omega \right)u_{\eta }\left(\text{r}\right){|}^{2}\rangle , \end{array}$$

where the angle brackets indicate averaging over random inputs where relevant. We can rewrite Equation 23 as


(24) $$\begin{array}{l} P\left(\text{r},\omega \right)=\langle \underset{\eta }{\sum }T(k_{\eta },\omega )\phi_{sn}\left(k_{\eta },\omega \right)u_{\eta }\left(\text{r}\right)\\ \;\times \underset{\mu }{\sum }T^{*}\left(k_{\mu },\omega \right)\phi_{sn}^{*}\left(k_{\mu },\omega \right)u_{\mu }^{*}(\text{r})\rangle , \end{array}$$

(25) $$\begin{array}{l} \;=\underset{\eta \mu }{\sum }T\left(k_{\eta },\omega \right)T^{*}\left(k_{\mu },\omega \right)u_{\eta }\left(\text{r}\right)u_{\mu }^{*}\left(\text{r}\right)\\ \;\times \langle \phi_{sn}\left(k_{\eta },\omega \right)\phi_{sn}^{*}\left(k_{\mu },\omega \right)\rangle , \end{array}$$

with


(26) $$\begin{array}{l} \phi_{sn}\left(k_{\eta },\omega \right)=\int \phi_{sn}\left(\text{r},\omega \right)u_{\eta }\left(\text{r}\right)d\text{r}, \end{array}$$

where the integral extends over the cortical surface.

In the case of random-phase inputs, which have been widely used to reproduce experimental spectra (Robinson et al., 1998, 2018; Gabay et al., 2018), the final average satisfies $\langle \phi_{sn}\left(k_{\eta },\omega \right)\phi_{sn}^{*}\left(k_{\mu },\omega \right)\rangle =|\phi_{sn}\left(k_{\eta },\omega \right){|}^{2}\delta_{\eta \mu }$, so


(27) $$\begin{array}{l} P\left(\text{r},\omega \right)=\underset{\eta }{\sum }|T\left(k_{\eta },\omega \right){|}^{2}|u_{\eta }\left(\text{r}\right){|}^{2}|\phi_{sn}\left(k_{\eta },\omega \right){|}^{2}, \end{array}$$

in the random-phase case. When integrated over **r** to get the total power spectrum, the orthonormality of the eigenfunctions implies


(28) $$\begin{array}{l} P\left(\omega \right)=\int P\left(\text{r},\omega \right)d\text{r}=\underset{\eta }{\sum }|T\left(k_{\eta },\omega \right){|}^{2}||\phi_{sn}\left(k_{\eta },\omega \right){|}^{2}. \end{array}$$

## 3 Results

In this section we first use our NFT to derive expressions for the modal alpha frequencies, including the effects of nonuniform loop delays and gains, and then derive the topographical distribution of alpha power from the NFT equations. These results rely on expansions of time delays and gains in terms of the eigenfunctions discussed in Section 2. The details of the derivations are not required in order to follow the accompanying comparisons with experimentally observed features of the rhythms.

### 3.1 Modal eigenvalue effect on alpha frequencies (*k*-effect)

**Figure 3:**
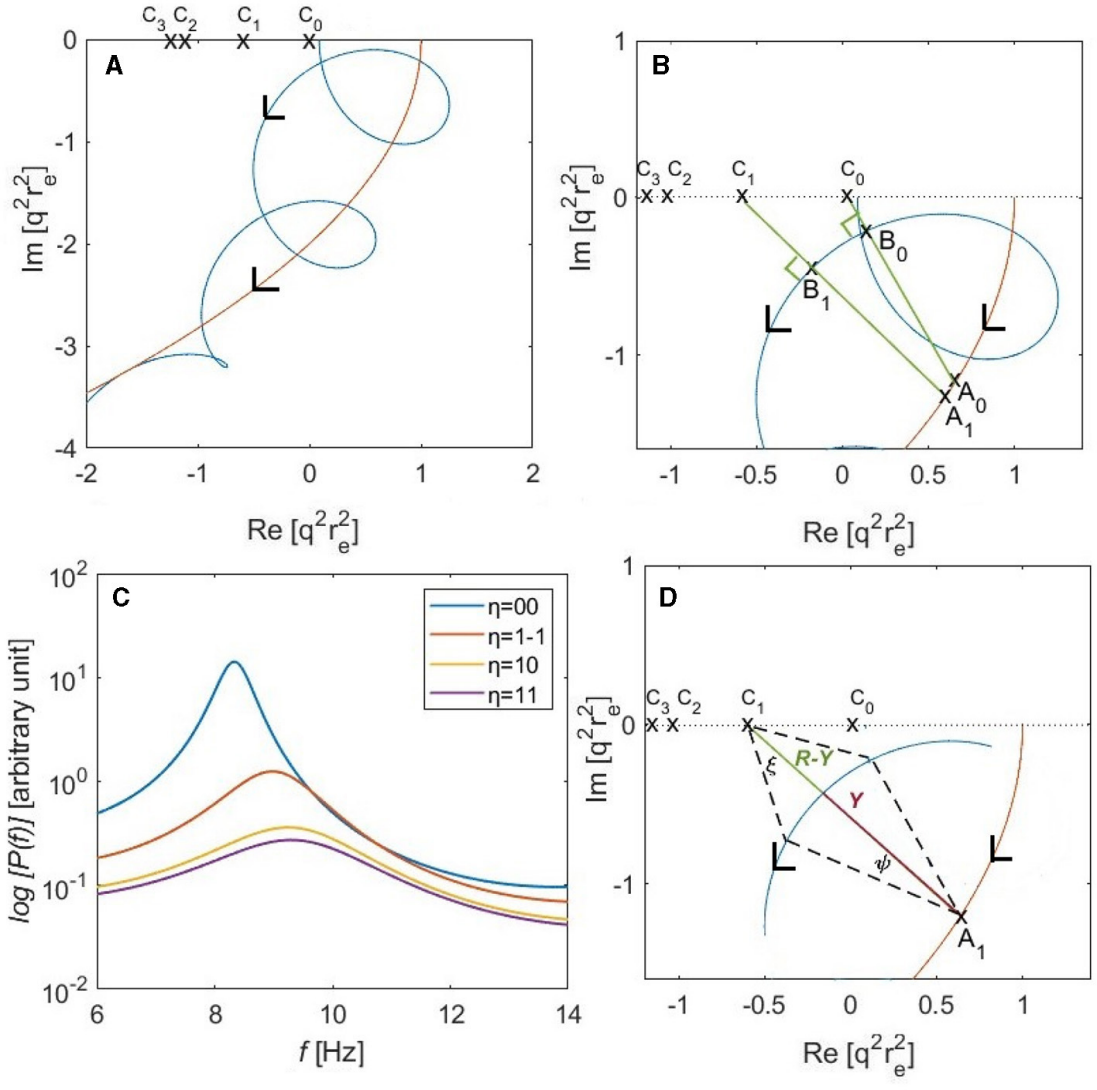
Analysis of modal alpha frequencies and power spectra for a convoluted brain hemisphere based on the parameters of Table 1 with *r*_*ee*_ = 60 mm. **(A)** The locus of $q^{2}\left(\omega \right)r_{ee}^{2}$ is shown as a blue curve in the complex plane according to Equation 29, with *ω* = 0 on the real axis and increasing in the direction of the arrow. The red parabola corresponds to the leading quadratic term in parentheses on the right of Equation 29 and C_0_, C_1_, C_2_, and C_3_ show $-k_{\eta }^{2}r_{ee}^{2}$ for *η* = 00, 1-1, 10, and 11, respectively. **(B)** Expanded view of $q^{2}\left(\omega \right)r_{ee}^{2}$ at low frequencies. **(C)** Individual modal spectra for the first four eigenmodes, *η* = 00, 1-1, 10, and 11, for the gain parameters in Table 1. A logarithmic scale is used to show the latter three spectra more clearly. **(D)** Geometry used to determine half-power points in Equation 75; the angle ξ approaches 45° at the half-power points when |*R* − *Y*|≪*Y*.

In this section, we use NFT described in the previous section to estimate the offset of each mode's alpha frequency from that of the lowest mode. Starting from Equation 15, and based on the assumption that the corticothalamic loop delay is uniform over the brain, we can write *τ*_0_ = *τ*_*es*_ + *τ*_*se*_, whence


(29) $$\begin{array}{l} q^{2}\left(\omega \right)r_{ee}^{2}={\left(1-\frac{i\omega }{\gamma_{ee}}\right)}^{2}-\frac{LG_{ee}}{1-LG_{ei}}-\frac{\left(G_{ese}+G_{esre}L\right)L^{2}e^{i\omega \tau_{0}}}{\left(1-LG_{ei}\right)\left(1-G_{srs}L^{2}\right)}, \end{array}$$

(30) $$\begin{array}{l} \;=1-\frac{\omega^{2}}{\gamma_{ee}^{2}}-\frac{2i\omega }{\gamma_{ee}}-X\left(\omega \right)-Y\left(\omega \right)e^{i\omega \tau_{0}}, \end{array}$$

(31) $$\begin{array}{l} \;X\left(\omega \right)=\frac{LG_{ee}}{1-LG_{ei}}, \end{array}$$

(32) $$\begin{array}{l} \;Y\left(\omega \right)=\frac{\left(G_{ese}+G_{esre}L\right)L^{2}}{\left(1-LG_{ei}\right)\left(1-G_{srs}L^{2}\right)}, \end{array}$$

where both *X*(*ω*) and *Y*(*ω*) decrease in magnitude at large *ω*. The leading quadratic term on the right of Equation 29 describes a parabola in the complex $q^{2}\left(\omega \right)r_{ee}^{2}$ plane, and the cyclic term in Equation 29 causes the loops shown in Figure 3A; the points C_0_, C_1_, C_2_, and C_3_ lie at $q^{2}\left(\omega \right)r_{ee}^{2}=-k_{\eta }^{2}r_{ee}^{2}$ for *η* = 00, 1-1, 10, and 11, respectively. Figure 3B shows a part of locus of $q^{2}\left(\omega \right)r_{ee}^{2}$ near the alpha frequency of the $k_{\eta }^{2}=0$ mode, which is the spatially uniform global mode with eigenfrequency *ω*_0_. The points A_0_ and A_1_ on the parabola are the centers of arcs of radii shown by the line segments |A_0_B_0_| and |A_1_B_1_|, at the spectral peaks of the eigenmodes *η* = 00 and *η* = 1-1, respectively. The length of the segment |C_*η*_B_*η*_| is the shortest distance from point C_*η*_ to the arc of radius |A_*η*_B_*η*_| centered at A_*η*_, while *θ*_*η*_ is the angle formed between |A_*η*_C_*η*_| and the imaginary axis. The angles at B_*η*_ in Figure 3B are right angles because it lies on the arc of radius |A_*η*_B_*η*_| centered at A_*η*_ such that the points A_0_, B_0_, and C_0_ are collinear.

We estimate modal frequencies in two stages, on the assumption that the system is driven by random-phase inputs so Equation 28 applies, with the transfer function given by Equation 14. First we calculate the uniform-mode frequency *ω*_00_ and then estimate the offsets from it to other modes' alpha-peak frequencies. Referring to Figure 3B, *ω*_00_ corresponds to the point at which $\left|q^{2}\left(\omega \right)r_{ee}^{2}\right|$ is closest to the pole at C_0_ = 0, which corresponds to the point A_0_ on the parabola.

The appearance of peaks in the power spectrum is mainly governed by the factor $L^{2}e^{i\omega \tau_{0}}$ in Equation 29, which causes the final denominator in Equation 14 to alternately increase and decrease in magnitude, with a period in *ω* of ~2π/*τ*_0_. Aside from the peak at *ω* = 0, the next peak thus occurs when *i*ωτ**_0_ ≈ 2π. For *ω* ≪ *α*, *β*, *γ*, this gives the cyclic frequency (Robinson et al., 2002).


(33) $$\begin{array}{l} f_{0}\approx {\left[\tau_{0}+\frac{2}{\alpha }+\frac{2}{\beta }\right]}^{-1}, \end{array}$$

where *τ*_0_ ≈ 80 ms is the mean corticothalamic time delay.

Our next step is to find the frequency offset *ω*_*η*_ − *ω*_00_ with the aim of being able to combine these estimates with experimentally observed *ω*_00_ to avoid the approximations in the previous paragraph and obtain more accurate results. If we again refer to Figure 3B, *ω*_*η*_ is the frequency at which the points A_*η*_, B_*η*_, and C_*η*_ are collinear. If we neglect the small offset between A_0_ and A_*η*_ relative to the distance between C_0_ and C_*η*_, the extra angle rotated by the vector |A_*η*_B_*η*_| relative to |A_0_B_0_| is


(34) $$\begin{array}{l} \theta_{\eta }-\theta_{00}\approx \left(\omega_{\eta }-\omega_{00}\right)\tau_{0}. \end{array}$$

The point A_*η*_ has coordinates


(35) $$\begin{array}{l} x_{\eta }=1-\frac{\omega_{\eta }^{2}}{\gamma_{ee}^{2}}-X\left(\omega_{\eta }\right), \end{array}$$

(36) $$\begin{array}{l} y_{\eta }=-\frac{2\omega_{\eta }}{\gamma_{ee}}. \end{array}$$

So, we find


(37) $$\begin{array}{l} \tan\theta_{0}=\frac{x_{0}}{\left|y_{0}\right|}, \end{array}$$

(38) $$\begin{array}{l} \tan\theta_{\eta }=\frac{x_{\eta }+k_{\eta }^{2}r_{ee}^{2}}{\left|y_{\eta }\right|}. \end{array}$$

Then, using Equations 34–38, we find


(39) $$\begin{array}{l} \omega_{\eta }-\omega_{00}=\frac{1}{\tau_{0}}\left[\tan^{-1}\left(\frac{x_{\eta }+k_{\eta }^{2}r_{ee}^{2}}{\left|y_{\eta }\right|}\right)-{\text{tan}}^{-1}\left(\frac{x_{0}}{\left|y_{0}\right|}\right)\right]\approx \frac{2\gamma_{ee}r_{ee}^{2}}{A_{S}}. \end{array}$$

Here, the rightmost expression is obtained by approximating the inverse tangent functions by their arguments, setting *x*_*η*_ = *x*_0_ and *y*_*η*_ = *y*_0_, and noting that in the limit of a spherical cortex of radius *R*_*S*_ and area *A*_*S*_ one has $k_{\eta }^{2}R_{S}^{2}=2$ for the three lowest nonuniform modes. From this approximation, we see that splitting is greatest for small brains with fast, long-range axons (i.e., *A*_*S*_ small, *γ*_*ee*_ and *r*_*ee*_ large). These results can be further refined by noting that Robinson et al. (2016) found $k_{1-1}^{2}R_{S}^{2}=1.2$, $k_{10}^{2}R_{S}^{2}=2.2$, $k_{11}^{2}R_{S}^{2}=2.6$, and *A*_*S*_ = 0.070 m^2^ for the Freesurfer group-average hemispheric surface used in their work and the present analysis. These parameters, together with those in Table 1, imply typical alpha splittings of ~ 1 Hz, which accords with observations (Chiang et al., 2008, 2011).

Now, Equation 39 is transcendental because *x*_*η*_ and *y*_*η*_ are functions of *ω*_*η*_ via Equations 35, 36. But it can be solved iteratively as follows: (i) On the right hand side, set $\omega_{\eta }=\omega_{00}=\omega_{\eta }^{\left(0\right)}$, $y_{\eta }=y_{0}=y_{\eta }^{\left(0\right)}$, and $x_{\eta }=x_{0}=x_{\eta }^{\left(0\right)}$, where the superscript indicates the iteration step. (ii) Solve to obtain new estimates for $\omega_{\eta }^{\left(1\right)}$ and the corresponding $x_{\eta }^{\left(1\right)}$ and $y_{\eta }^{\left(1\right)}$. (iii) Set $\omega_{\eta }=\omega_{\eta }^{\left(1\right)}$, $x_{\eta }=x_{\eta }^{\left(1\right)}$, and $y_{\eta }=y_{\eta }^{\left(1\right)}$ on the right hand side. (iv) Repeat steps (ii) and (iii) to obtain successive estimates of $\omega_{\eta }^{\left(j\right)}$ for *j* = 2, 3, … until convergence is achieved. Noting that *ω*_00_*τ*_0_ ≈ 2π places the upper bound


(40) $$\begin{array}{l} \omega_{\eta }<\omega_{00}\left[\frac{5}{4}-\frac{1}{2\pi }\tan^{-1}\left(\frac{x_{0}}{\left|y_{0}\right|}\right)\right], \end{array}$$

on *ω*_*η*_, so *ω*_*η*_ < 3*ω*_00_/2, with *ω*_*η*_ < 5*ω*_00_/4 if *x*_0_ > 0. This means starting with *f*_0_ = 8.1 Hz (Gabay et al., 2018) the modal alpha frequency for the *l* = 1 and *l* = 2 modes should not exceed 10.1 Hz, which agrees with the results of Gabay et al. (2018).

Equation 33 yields *f*_0_ = 7.7 Hz, which has a significant offset from the numerical value of 8.1 Hz obtained by Gabay et al. (2018) for the relevant values of *α*, *β*, and *γ*; this is the result of the approximations made in obtaining Equation 33. Thus, we focus on the offsets obtained from Equation 39 on the basis that these are independent of the baseline estimate from Equation 33, which can be replaced by an experimental value of *ω*_00_ if desired. We find that the frequency offsets obtained from Equation 39 are 1.4, 1.8, and 1.9 Hz for the 1-1, 10, and 11 modes, respectively, which are within ~0.1 Hz of those obtained from the full numerical analysis of Gabay et al. (2018), which were 1.5, 1.7, and 1.8 Hz. Notably, the differences between the frequencies of the *l* = 1 modes are much smaller than their offsets from the 00 mode for these parameters.

Figure 3C shows the individual modal spectra of the first four eigenmodes for the parameters shown in Table 1, which differ somewhat from those used by Gabay et al. (2018). The *l* = 0 eigenmode has a greater overall power than the *l* = 1 modes. The alpha frequency of the *u*_1 − 1_ mode is shifted upward by around 0.7 Hz from that of the uniform mode, and the frequency offsets for the *u*_10_ and *u*_11_ modes by around 0.9 and 1 Hz, respectively. This figure illustrates the *k*-effect on the alpha frequency shift. The next section presents another effect on the alpha frequency, which is the non-uniformity in the corticothalamic loop delay (*τ*-effect).

### 3.2 Nonuniform corticothalamic loop delay (*τ*-effect)

We must still estimate the effects of non-uniformities in loop delays on modal alpha frequencies. In this section, we do this on the assumption that changes in frequency are small relative to the alpha frequency in the *l* = 0 mode and treat them as additive. Moreover, we approximate the loop delay for each mode by the expectation value of the local delay *τ*(**r**) in that mode when we generalize *τ* to have an **r** dependence. This corresponds to neglecting mode coupling (O'Connor and Robinson, 2004) by non-uniformities in *τ*(**r**), which is self-consistent so long as these non-uniformities are not too large.

#### 3.2.1 General analysis

To first order in perturbations relative to the mean value, we can generalize *τ*_0_ in Equations 29, 30 to the form


(41) $$\begin{array}{l} \tau \left(\text{r}\right)\approx \tau_{0}+\tau_{1}\left(\text{r}\right), \end{array}$$

where *τ*_0_ is the mean time delay, and *τ*_1_(**r**) is the perturbed time delay. If we then consider the term containing the *τ*(**r**) in Equation 30, then its expectation value for the corresponding mode *η* is


(42) $$\begin{array}{l} \langle \eta |e^{i\omega \tau \left(\text{r}\right)}|\eta \rangle \approx \langle \eta |e^{i\omega \tau_{0}}\left[1+i\omega \tau_{1}\left(\text{r}\right)\right]|\eta \rangle , \end{array}$$

(43) $$\begin{array}{l} \;=e^{i\omega \tau_{0}}\left[1+i\omega \langle \eta |\tau_{1}\left(\text{r}\right)|\eta \rangle \right], \end{array}$$

(44) $$\begin{array}{l} \;\approx \text{exp}[i\omega \left(\tau_{0}+\langle \eta |\tau_{1}\left(\text{r}\right)|\eta \rangle \right], \end{array}$$

(45) $$\begin{array}{l} \;=\text{exp}\left[i\omega \langle \eta |\tau \left(\text{r}\right)|\eta \rangle \right]. \end{array}$$

Here we have ignored second-order terms and have used the Dirac notation


$$\begin{array}{l} \langle \eta |g\left(\text{r}\right)|\eta \rangle =\int |u_{\eta }\left(\text{r}\right){|}^{2}g\left(\text{r}\right)d^{2}\text{r}, \end{array}$$

to represent the expectation value of an arbitrary square-integrable function *g*(**r**) in the mode *η*.

Assuming that other terms in Equation 30 are little changed from their values in the uniform mode, the phase of the cyclic term in Equation 30 at the modal alpha peak will be very nearly the same as in the uniform mode. Therefore, if we write the alpha frequency of mode *η* as *ω*_*η*_ = *ω*_00_ + Δ*ω*_*η*_, to first order it will satisfy


(46) $$\begin{array}{l} \left(\omega_{0}+\text{Δ}\omega_{\eta }\right)\langle \eta |\tau \left(\text{r}\right)|\eta \rangle =\omega_{0}\tau_{0}, \end{array}$$

whence


(47) $$\begin{array}{l} \omega_{0}\tau_{0}+\text{Δ}\omega_{\eta }\tau_{0}+\omega_{0}\langle \eta |\tau_{1}\left(\text{r}\right)|\eta \rangle =\omega_{0}\tau_{0}, \end{array}$$

again to first order. Hence,


(48) $$\begin{array}{l} \text{Δ}\omega_{\eta }=-\omega_{0}\frac{\langle \eta |\tau_{1}\left(\text{r}\right)|\eta \rangle }{\tau_{0}}. \end{array}$$

This shift must be added to the corresponding frequency found in Section 3.1, since we are working only to first order.

#### 3.2.2 Spherical cortex

To estimate the *τ*-effect on the alpha frequency we need to calculate the expectation values in Equation 45. To illustrate the ideas, we start with the approximation of a spherical cortex; while the following subsection treats the general case of a convoluted cortex.

Assuming a spherical cortex, the spatial eigenmodes will be the spherical harmonics (Maximon, 2010), hence, we can expand the time delay for a spherical brain surface in terms of the spherical harmonics, giving


(49) $$\begin{array}{l} \tau \left(\text{r}\right)=\underset{lm}{\sum }a_{lm}Y_{lm}, \end{array}$$

(50) $$\begin{array}{l} \;=a_{00}Y_{00}+\overset{1}{\underset{m=-1}{\sum }}a_{1m}Y_{1m}+\overset{2}{\underset{m=-2}{\sum }}a_{2m}Y_{2m}+\ldots , \end{array}$$

where we have omitted arguments of the *Y*_*lm*_ for compactness and the *a*_*lm*_ are the real-valued expansion coefficients


(51) $$\begin{array}{l} a_{lm}=\int \tau \left(\text{r}\right)Y_{lm}d\text{Ω}, \end{array}$$

where *d*Ω = sin *ϑ* *d*ϑ* d*φ** in spherical coordinates.

If we consider only the first nine eigenmodes (*l* ≤ 2), we get


(52) $$\begin{array}{l} \tau \left(\text{r}\right)=\overset{2}{\underset{l=0}{\sum }}\overset{l}{\underset{m=-l}{\sum }}a_{lm}Y_{lm}=a_{00}Y_{00}+\tau_{1}\left(\text{r}\right), \end{array}$$

where


(53) $$\begin{array}{l} \tau_{1}\left(\text{r}\right)\approx \overset{1}{\underset{m=-1}{\sum }}a_{1m}Y_{1m}+\overset{2}{\underset{m=-2}{\sum }}a_{2m}Y_{2m}. \end{array}$$

We then have


(54) $$\begin{array}{l} {\langle \tau_{1}\rangle }_{\eta }=\langle \eta |\tau_{1}\left(\text{r}\right)|\eta \rangle . \end{array}$$

If we now let *η* denote a mode labeled *LM*, we can then write


(55) $$\begin{array}{l} {\langle \tau_{1}\rangle }_{LM}=\langle LM|\tau_{1}\left(\text{r}\right)|LM\rangle , \end{array}$$

(56) $$\begin{array}{l} \;=\int Y_{LM}^{*}\tau_{1}\left(\text{r}\right)Y_{LM}d\text{Ω}, \end{array}$$

(57) $$\begin{array}{l} \;=\int Y_{LM}^{*}\left(\overset{2}{\underset{l=0}{\sum }}\overset{l}{\underset{m=-l}{\sum }}a_{lm}Y_{lm}\right)Y_{LM}d\text{Ω}, \end{array}$$

(58) $$\begin{array}{l} \;=\overset{2}{\underset{l=0}{\sum }}\overset{l}{\underset{m=-l}{\sum }}I_{lm}^{LM}a_{lm}, \end{array}$$

where the individual contribution to the delay is


(59) $$\begin{array}{l} I_{lm}^{LM}=\int Y_{LM}^{*}Y_{lm}Y_{LM}d\text{Ω}. \end{array}$$

Hence,


(60) $$\begin{array}{l} {\langle \tau_{1}\rangle }_{\eta }=a_{1-1}I_{1-1}^{\eta }+a_{10}I_{10}^{\eta }+a_{11}I_{11}^{\eta }+a_{2-2}I_{2-2}^{\eta }\\ \;+a_{2-1}I_{2-1}^{\eta }+a_{20}I_{20}^{\eta }+a_{21}I_{21}^{\eta }+a_{22}I_{22}^{\eta }. \end{array}$$

Note that there is no 00 term in Equation 60 because this is already included in the mean value of *τ*.

The above integrals can be evaluated analytically in terms of the Wigner 3j symbols, which also arise in treating coupled angular momenta in quantum systems, such that in spherical coordinates


(61) $$\begin{array}{l} \int_{0}^{2\pi }\int_{0}^{\pi }Y_{L_{1}M_{1}}\left(\vartheta ,\varphi \right)Y_{lm}\left(\vartheta ,\varphi \right)Y_{L_{2}M_{2}}\left(\vartheta ,\varphi \right)\sin\vartheta d\vartheta d\varphi \\ \;={\left[\frac{\left(2L_{1}+1\right)\left(2l+1\right)\left(2L_{2}+1\right)}{4\pi }\right]}^{1/2}\left(\begin{array}{ccc} L_{1} & l & L_{2}\\ 0 & 0 & 0 \end{array}\right)\left(\begin{array}{ccc} L_{1} & l & L_{2}\\ M_{1} & m & M_{2} \end{array}\right), \end{array}$$

where $\left(\begin{array}{ccc} L_{1} & l & L_{2}\\ 0 & 0 & 0 \end{array}\right)$ and $\left(\begin{array}{ccc} L_{1} & l & L_{2}\\ M_{1} & m & M_{2} \end{array}\right)$ are 3j symbols (Maximon, 2010). Therefore, we can find 〈_*τ*_1_〉*η*_ for all the eigenmodes in terms of the coefficients *a*_*lm*_. Table 2 shows the values of the integrals $I_{lm}^{LM}$ in Equation 59 for the first nine spherical harmonics. Because the $Y_{LM}^{*}Y_{LM}$ part of the integrand in Equation 59 is always even in *ϑ* and *φ*, only an even middle part *Y*_*lm*_ can give a nonzero result for the sphere. Note that the *l* = 0 mode is not affected by the perturbations from the other eigenmodes, and its corresponding integral gives zero by definition; hence, all the integrals in Equation 60 vanish except the $I_{20}^{\eta }$, as seen in Table 2. Those integrals are identically zero because they involve integration of an antisymmetric function over a symmetric domain in *ϑ* and/or *φ*. Therefore, in the spherical case, Equation 60 can be simplified to


(62) $$\begin{array}{l} {\langle \tau_{1}\rangle }_{\eta }=a_{20}I_{20}^{\eta }. \end{array}$$

**Table 2:** Integrals used in expansions of quantities on a spherical cortical surface in standard spherical coordinates.

**Mode affected (*Y*_*LM*_)**	**Perturbation component (** ***Y***_*****lm*****_ **)**							
	*Y*_1 − 1_	*Y*_10_	*Y*_11_	*Y*_2 − 2_	*Y*_2 − 1_	*Y*_20_	*Y*_21_	*Y*_22_
*Y*_00_	0	0	0	0	0	0	0	0
*Y*_1 − 1_	**0**	**0**	**0**	0	0	− 0.126	0	0
*Y*_10_	**0**	**0**	**0**	0	0	0.252	0	0
*Y*_11_	**0**	**0**	**0**	0	0	− 0.126	0	0
*Y*_2 − 2_	0	0	0	0	0	− 0.180	0	0
*Y*_2 − 1_	0	0	0	0	0	0.090	0	0
*Y*_20_	0	0	0	0	0	0.180	0	0
*Y*_21_	0	0	0	0	0	0.090	0	0
*Y*_22_	0	0	0	0	0	− 0.180	0	0

Values of integrals $I_{lm}^{LM}$ in Equation 59 for the first nine spherical harmonics. The rows in the body of the table correspond to the spherical eigenmodes *Y*_*LM*_ (leftmost column) affected by the perturbation components from the eigenmodes *Y*_*lm*_ (top row). The bold numbers correspond to the effect of the perturbations from each of the *l* = 1 modes on each of the *L* = 1 modes.

#### 3.2.3 Convoluted cortex

As noted in Section 2.1.2, the human cerebral cortex can be approximated as a highly convoluted sheet, albeit with a geometry that makes computational analysis and visualization difficult. However, the methods described there enable a mapping to a spherical surface that allows a coordinate system to be established for use in calculation and visualization.

On a sphere, the *Y*_10_ and *Y*_11_ modes have nodal lines that intersect at the north and south poles (i.e., *ϑ* = 0, 180°, respectively). In our framework, we define the north pole on the inflated sphere to be the intersection of the corresponding nodal lines; however, these points do not occur at the north and south poles of the Freesurfer coordinate system seen in Figure 2B. Accordingly, we define a new coordinate system by rotating the Freesurfer coordinate system on the inflated cortex to place the north pole at the appropriate intersection, as seen in Figure 2C. The rotated coordinate system is more efficient because (i) in the limiting case that the cortex is actually a sphere (i.e., no inflation required), the cortical eigenmodes will correspond exactly to the spherical harmonics, rather than linear combinations thereof, which greatly simplifies calculations; and (ii) the low-order convoluted modes are closely related to the spherical harmonics (Robinson et al., 2016; Gabay and Robinson, 2017), so corresponding simplifications are also attained in this case. In contrast, if we were to use the Freesurfer coordinate system, the eigenmodes even of a spherical cortex would no longer be the spherical harmonics, but a rotated version of them, such that each spherical eigenmode $Y_{lm}^{\prime }$ is a linear combination of the spherical harmonics $Y_{lm^{\prime }}$ where *m*′ varies between − *l* and *l*. Consequently, evaluating the integrals of Equation 59 for the corresponding first nine spherical eigenmodes yields (Table 3) in Freesurfer coordinates, which has many more nonzero entries than Table 2. Interestingly, similarly to the spherical cortex case in Section 3.2.2, the perturbation components from the *l* = 1 modes have no effect on these nine eigenmodes. This is due the fact that coordinate rotation preserves antisymmetry for each *l* = 1 mode, but not for individual *l* = 2 modes, which yields many nonzero entries in the table.

**Table 3:** Integrals used in expansions of quantities on a spherical cortical surface using Freesurfer coordinates.

**Mode affected ($Y_{LM}^{\prime }$)**	**Perturbation component (** ***Y***_*****lm*****_ **)**							
	$Y_{1-1}^{\prime }$	$Y_{10}^{\prime }$	$Y_{11}^{\prime }$	$Y_{2-2}^{\prime }$	$Y_{2-1}^{\prime }$	$Y_{20}^{\prime }$	$Y_{21}^{\prime }$	$Y_{22}^{\prime }$
$Y_{00}^{\prime }$	0	0	0	0	0	0	0	0
$Y_{1-1}^{\prime }$	**0**	**0**	**0**	0.160	− 0.180	− 0.033	0.040	− 0.078
$Y_{10}^{\prime }$	**0**	**0**	**0**	− 0.150	0.065	0.033	− 0.140	− 0.130
$Y_{11}^{\prime }$	**0**	**0**	**0**	− 0.004	0.110	0.0004	0.100	0.200
$Y_{2-2}^{\prime }$	0	0	0	0.010	− 0.095	0.0002	− 0.120	− 0.100
$Y_{2-1}^{\prime }$	0	0	0	− 0.0003	− 0.180	− 0.002	0.014	0.011
$Y_{20}^{\prime }$	0	0	0	0.002	0.180	0.002	− 0.012	0.006
$Y_{21}^{\prime }$	0	0	0	− 0.006	0.085	− 0.004	0.130	− 0.095
$Y_{22}^{\prime }$	0	0	0	− 0.005	0.009	0.004	− 0.014	0.180

Values of integrals $I_{lm}^{LM}$ in Equation 59 in Freesurfer coordinates for the first nine spherical eigenmodes, such that the rows in the body of the table correspond to the spherical eigenmodes $Y_{LM}^{\prime }$ (leftmost column) affected by the perturbation components from the eigenmodes $Y_{lm}^{\prime }$ (top row). The bold numbers correspond to the effect of the perturbations from each of the *l* = 1 modes on each of the *L* = 1 modes.

Returning to the general case of real eigenmodes *u*_*lm*_, these modes reproduce the real spherical harmonics as the amount of cortical convolution is artificially reduced via a continuous mapping from convoluted cortex to sphere via intermediate shapes (Robinson et al., 2016; Gabay et al., 2018). We can express *τ*(**r**) in terms of the eigenmodes such that


(63) $$\begin{array}{l} \tau \left(\text{r}\right)=\underset{lm}{\sum }a_{lm}u_{lm}, \end{array}$$

(64) $$\begin{array}{l} \;=a_{00}u_{00}+\overset{1}{\underset{m=-1}{\sum }}a_{1m}u_{1m}+\overset{2}{\underset{m=-2}{\sum }}a_{2m}u_{2m}+\ldots , \end{array}$$

where the coefficients *a*_*lm*_ are real. Hence,


(65) $$\begin{array}{l} {\langle \tau_{1}\left(\text{r}\right)\rangle }_{LM}=\int u_{LM}^{*}\left(\underset{lm}{\sum }a_{lm}u_{lm}\right)u_{LM}d\text{Ω}, \end{array}$$

(66) $$\begin{array}{l} \;=\underset{lm}{\sum }a_{lm}J_{lm}^{LM}, \end{array}$$

where


(67) $$\begin{array}{l} J_{lm}^{LM}=\int |u_{LM}{|}^{2}u_{lm}d\text{Ω}, \end{array}$$

since the eigenmodes here are real. We can evaluate these integrals numerically, and have checked the accuracy of our code in the spherical case. Table 4 shows the $J_{lm}^{LM}$ from Equation 67 for the first nine modes, corresponding to those in Tables 2, 3 in the spherical limit. Notably, Table 4 is full except for its 00 row, unlike Tables 2, 3, because of symmetry breaking by the convoluted cortical surface, except that the uniform mode is unaffected because the relevant integrals vanish identically. Some integrals are much smaller than others, the larger ones being of order 0.1, as in Table 2, whereas the smaller are of order 0.001. Those integrands with approximately odd front-back parity tend to give near-zero integrals, as for a sphere where this antisymmetry is exact. Indeed, our numerical analysis reproduces the analytic spherical result in the relevant limit. The effects on the first four eigenmodes caused by the first four perturbation components are represented by bold entries in Tables 2–4. Hence, focusing only on this section of each table, we notice that the integrals vanish in the spherical limit for both unrotated and rotated cases; however, this part of the table is full for the convoluted cortex as a result of symmetry breaking.

**Table 4:** Integrals used in expansions of quantities on the nominal Freesurfer convoluted cortical surface using Freesurfer coordinates.

**Mode affected**	**Perturbation component (** ***u***_*****lm*****_ **)**							
*u*_*LM*_	*u*_1 − 1_	*u*_10_	*u*_11_	*u*_2 − 2_	*u*_2 − 1_	*u*_20_	*u*_21_	*u*_22_
*u*_00_	0	0	0	0	0	0	0	0
*u*_1 − 1_	**0.008**	**0.050**	− **0.110**	− 0.220	0.160	0.084	− 0.025	0.0004
*u*_10_	− **0.045**	− **0.025**	**0.089**	0.170	− 0.022	0.011	− 0.210	0.160
*u*_11_	− **0.018**	− **0.002**	**0.082**	0.020	− 0.130	− 0.056	0.160	− 0.120
*u*_2 − 2_	− 0.027	− 0.001	− 0.180	0.009	0.020	− 0.082	− 0.022	0.050
*u*_2 − 1_	0.075	− 0.022	0.052	− 0.011	0.130	0.034	− 0.012	− 0.044
*u*_20_	0.058	0.092	0.015	− 0.076	− 0.003	0.008	0.095	− 0.090
*u*_21_	0.072	0.030	0.109	0.086	− 0.094	0.075	− 0.057	0.030
*u*_22_	− 0.088	− 0.030	− 0.045	0.111	− 0.098	− 0.065	− 0.019	0.044

Values of integrals $J_{lm}^{LM}$ in Equation 67 for the first nine eigenmodes on a convoluted cortex, such that the rows in the body of the table correspond to the eigenmodes *u*_*LM*_ (leftmost column) affected by the perturbation components from the eigenmodes *u*_*lm*_ (top row). The bold numbers correspond to the effect of the perturbations from each of the *l* = 1 modes on each of the *L* = 1 modes.

It has been argued that the main variation of the corticothalamic time delay within a hemisphere will be from front to back, with a lower value in the occipital region (Robinson et al., 2003; Chiang et al., 2008). Looking at Figure 2, the two modes that vary mainly front-to-back, as opposed to left-to-right, are *u*_1 − 1_ and *u*_10_ modes, whence we can approximate *τ*_1_ as a linear combination of these modes such that


(68) $$\begin{array}{l} \tau_{1}\left(\text{r}\right)=B\left[u_{1-1}\left(\text{r}\right)\cos\chi +u_{10}\left(\text{r}\right)\sin\chi \right], \end{array}$$

where χ is a mixing angle. We can then rewrite Equation 65 as


(69) $$\begin{array}{l} {\langle \tau_{1}\left(\text{r}\right)\rangle }_{LM}=-B[J_{1-1}^{LM}\cos\chi +J_{10}^{LM}\sin\chi ], \end{array}$$

where χ ≈ 30° in this case, and *B* is a positive coefficient.

**Figure 4:**
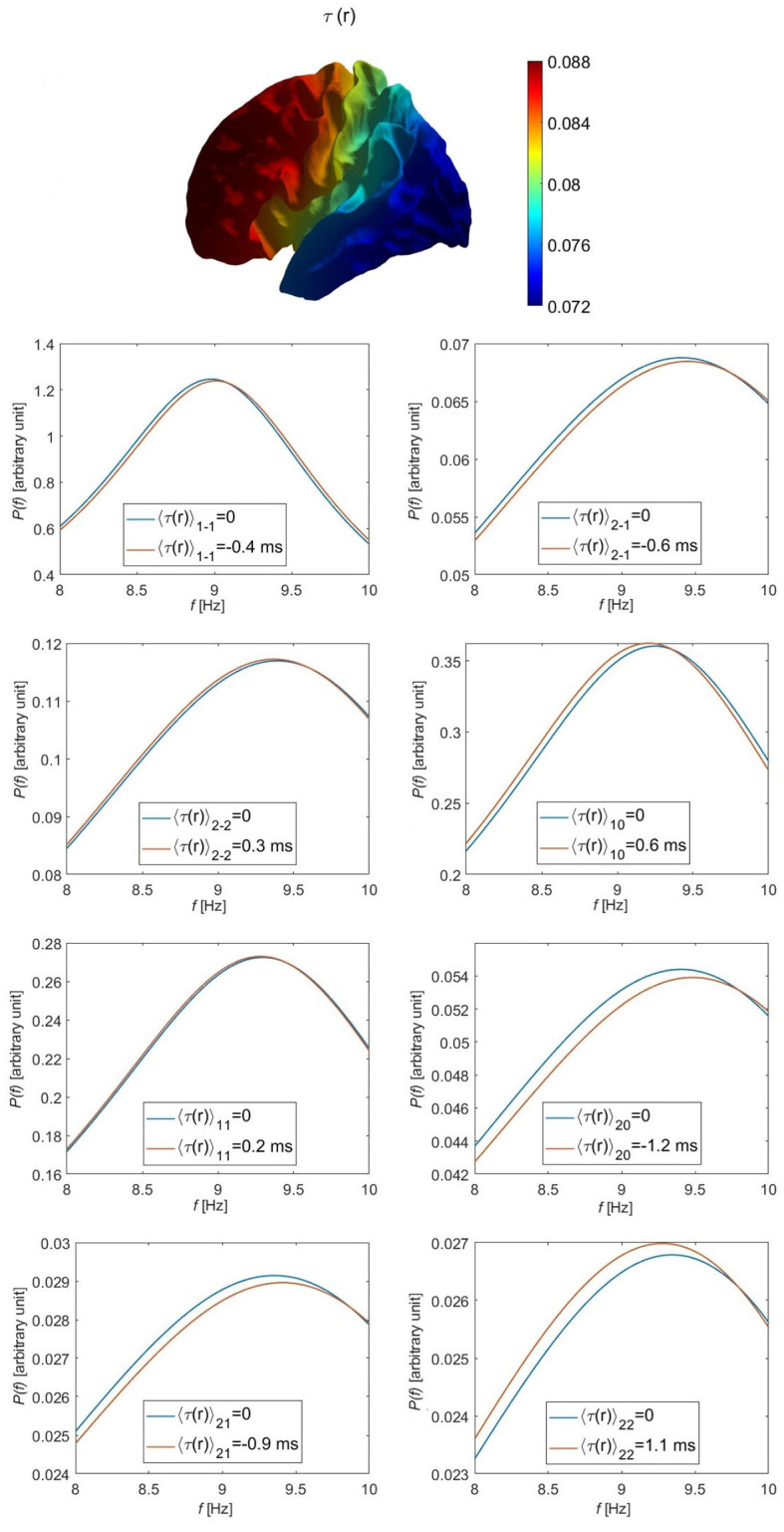
Individual modal spectra for the *l* = 1 and *l* = 2 eigenmodes, as labeled, including (red curves) and omitting (blue curves) the variation in the corticothalamic time delay *τ* for the parameters given in Table 1 and *τ*_1max_ = 8 ms (there is no effect on the *l* = 0 mode). The top leftmost figure shows the variation in the assumed corticothalamic time delay over the cortical surface.

We choose *B* in Equation 69 to give illustrative extremal values of + 8 and − 8 ms for *τ*_1_(**r**) at front and back of the head, respectively, (i.e., *τ*_1max_ = 0.1*τ*_0_). The numerical alpha frequency offset for each eigenmode is then estimated numerically by plotting the individual power spectra for each of the *l* = 1 and *l* = 2 eigenmodes when each eigenmode has its respective time delay, as seen in Figure 4. These numerical values are compared with their corresponding approximated values from Equation 48, as shown in Table 5. We find that *u*_11_ and *u*_2 − 2_ are the modes least affected by the variation of the time delay; *u*_1 − 1_ has a small alpha frequency shift of around 0.05 Hz from the original mode and a slightly higher power, while *u*_10_ has an alpha frequency shift of around − 0.05 Hz from the original mode and a slightly lower power. The *l* = 2 modes are the most affected, such that *u*_20_ has the highest alpha frequency shift of about 0.1 Hz. The average percentage difference between the numerical calculation of the alpha frequency shift shown in Figure 4 and the the corresponding approximated values in Table 6 is about 16%, with a smaller difference for the *l* = 1 modes than that for the *l* = 2 modes. The residual differences result from the omission of second order perturbation terms in going from Equation 42 to the approximation given by Equation 45.

**Table 5:** Values of the expected variation in corticothalamic time delay 〈*LM*|*τ*_1_(**r**)|*LM*〉 in mode *LM* affected by the perturbation from mode *lm* from Equation 65, and the corresponding approximated alpha frequency shift Δ*f*_app_ from Equation 48 as well as the numerical alpha frequency shift Δ*f*_num_ for each of the first nine eigenmodes when *τ*_1max_ = 8 ms.

***LM***	**〈*LM*|*τ*_1_(r)|*LM*〉(ms)**	***Δf*_app_ (Hz)**	***Δf*_num_ (Hz)**
00	0	0	0
1 − 1	− 0.4	0.04	0.05
10	0.6	− 0.06	− 0.05
11	0.2	− 0.02	− 0.02
2 − 2	0.3	− 0.03	− 0.03
2 − 1	− 0.7	0.07	0.05
20	− 1.2	0.12	0.08
21	− 1.0	0.10	0.07
22	1.1	− 0.11	− 0.08

**Table 6:** Parameters used in Figures.

**Case**	**Figures**	**Modes**	***r*_*ee*_ (mm)**	***G*_*es*_**	***G*_*se*_**	***a*_1 − 1_**	***a*_10_**	***a*_11_**
All	5–8	00	*	0.77	7.77	—	—	—
Default	5B	1-1	60	0.77	7.77	1	0	0
Small Splitting	5D, E	1-1	50	0.82	9.8	1	0	0
Large Splitting	5F, G	1-1	70	0.98	11.3	1	0	0
Increased Gain	5B, C	1-1	60	0.88	10.5	1	0	0
Single Alpha	6	1-1	50	0.82	9.8	− 0.08	0	0
Double Alpha	7	1-1,11	70	0.98	11.3	0.69	0	1.10
Mu	8B–D	10	50	0.82	9.8	0	− 0.09	0
Tau	8F–H	11	50	0.82	9.8	0	0	0.32

The 00 mode has the parameters listed in the row labeled All in every case, except that the asterisk indicates that the same value of *r*_*ee*_ is to be used as for the other modes in each case listed in subsequent rows. In other instances, the listed *l* = 1 modes have the parameters given. Parameters not shown are as in Table 1 and modes not listed are omitted from the case in question.

**Figure 5:**
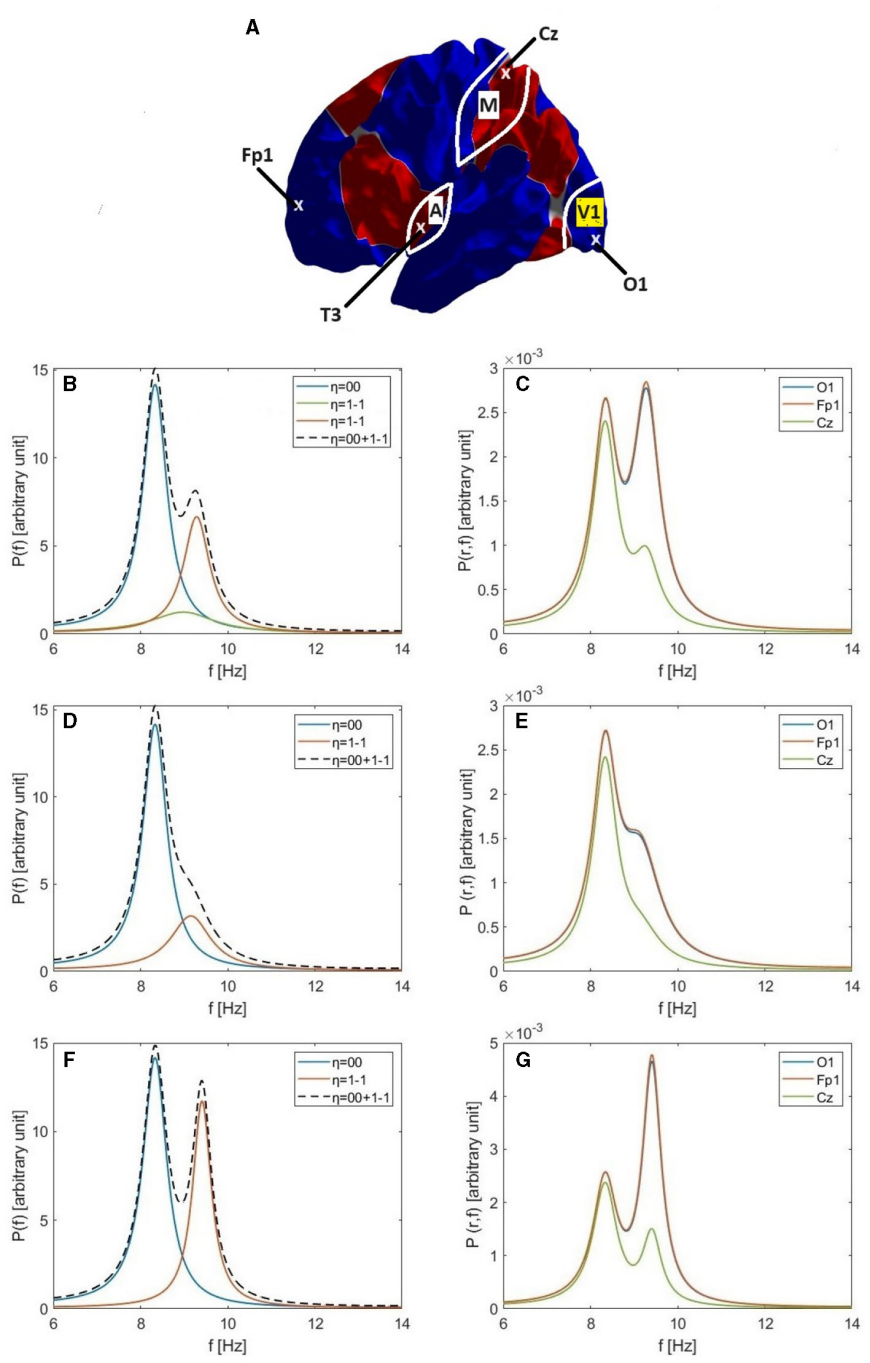
Single and double alpha spectral peaks. **(A)** Approximate locations of the O1, Fp1, Cz, and T3 electrodes; the locations of primary visual cortex (V1), primary auditory cortex (A), and primary motor cortex (M); and the nodal lines of the 1-1, 10, and 11 modes, which follow the boundaries between the colored regions. **(B)** Individual modal spectra for *η* = 00 (blue) and *η* = 1-1 (green) for the Default parameters in Table 6. The red curve shows the 1-1 spectrum for the Increased Gain case and the dashed curve is the resulting total spectrum *P*(*ω*). **(C)** The corresponding power spectra *P*(**r**, *ω*) at O1 (blue), Fp1 (red), and Cz (green) electrodes for the Increased Gain case. **(D)** Same as **(B)** but for the Small Splitting parameters in Table 6. **(E)** Same as **(C)** but for the Small Splitting parameters. **(F)** Same as **(B)** but for the Large Splitting parameters in Table 6. **(G)** Same as **(C)** but for the Large Splitting parameters.

For the above parameters, the largest shift in frequency caused by the variation in the time delay does not exceed 0.12 Hz; hence, to get the typically observed 1–2 Hz alpha frequency shift (Chiang et al., 2008, 2011) we would need to increase the assumed perturbation in the time delay to about 100% of its value in the uniform case, which is unrealistic. Hence, the *k*-effect is dominant over the *τ*-effect, causing shifts of about ~ 1 Hz between the uniform and the other brain modes. This implies that the *τ*-effect mechanism proposed by Robinson et al. (2003) and Chiang et al. (2008) is unlikely to account for the observed shifts. They argued that the largest effective values of *τ* would be of the same order as its maximal value, whereas we find here that cancelation of positive and negative contributions typically reduces it by an order of magnitude, as seen in the second column of Figure 5.

### 3.3 Sharpness of the alpha peak

The observability of a clear alpha rhythm is largely determined by the sharpness of the alpha spectral peak. From Equations 14, 25 the power spectrum of mode *η* can be expressed as


(70) $$\begin{array}{l} P_{\eta }\left(\text{r},\omega \right)=\frac{|u_{\eta }\left(\text{r}\right){|}^{2}|A\left(\omega \right){|}^{2}}{|k_{\eta }^{2}r_{ee}^{2}+q^{2}\left(\omega \right)r_{ee}^{2}{|}^{2}} \end{array}$$

where *A*(*ω*) is a function of *ω* resulted after substituting Equation 14 into Equation 25. The denominator in Equation 70 is equal to the square of the distance |A_*η*_C_*η*_| in Figure 3B.

If we let |A_*η*_C_*η*_| = *R*_*η*_ and |A_*η*_B_*η*_| = *Y* (here, *Y* is assumed to be mode-independent, so it only depends on the frequency), then at maximum alpha power


(71) $$\begin{array}{l} P_{\eta }\left(\text{r},\omega_{\alpha }\right)\approx \frac{|u_{\eta }\left(\text{r}\right){|}^{2}|A\left(\omega_{\alpha }\right){|}^{2}}{|R_{\eta }-Y{|}^{2}}. \end{array}$$

Likewise, the minimum alpha power is reached ~180° away on the $q^{2}\left(\omega \right)r_{ee}^{2}$ loop such that


(72) $$\begin{array}{l} P_{\eta }\left(\text{r},\omega_{\alpha }\right)\approx \frac{|u_{\eta }\left(\text{r}\right){|}^{2}|A\left(\omega_{\alpha }\right){|}^{2}}{|R_{\eta }+Y{|}^{2}}. \end{array}$$

Hence, we can define the quality factor ρ as the ratio between the maximum alpha power to the minimum alpha power at the opposite side of the loop; i.e.,


(73) $$\begin{array}{l} \rho_{\eta }\approx |\frac{R_{\eta }+Y}{R_{\eta }-Y}{|}^{2} \end{array}$$

The quality factor reflects the sharpness of the alpha peak. Hence, for sharp peaks where *Y* ≈ *R*_*η*_


(74) $$\begin{array}{l} \rho_{\eta }\approx \frac{4R_{\eta }^{2}}{{\left(R_{\eta }-Y\right)}^{2}}. \end{array}$$

We can also estimate the half width of the peak at half maximum by noting that the power is dominated by a factor proportional to $|k_{\eta }^{2}+q^{2}\left(\omega \right){|}^{-2}$, when this factor doubles, the power halves. Reference to Figure 3D shows that this occurs when the distance from the relevant pole increases to $\sim \sqrt{2}$ of its minimum value, so *ξ* ≈ 45° when |*R*_*η*_ − *Y*| ≪ *Y*. The cyclic phase *ωτ*_0_ thus differs from its value at the peak by an amount *ψ*, with


(75) $$\begin{array}{l} Y\sin\psi \approx |R_{\eta }-Y|. \end{array}$$

If δ*ω* is the half width at half maximum, then *ψ* ≈ *τ*_0_δ*ω*. If *ψ* is small, then


(76) $$\begin{array}{l} \delta \omega_{\eta }\approx \frac{1}{\tau_{0}}\frac{\left|R_{\eta }-Y\right|}{Y}, \end{array}$$

Our numerical results imply that a better approximation is obtained in the present case by multiplying Equation 76 by around 0.5, to give


(77) $$\begin{array}{l} \delta \omega_{\eta }\approx \frac{0.5}{\tau_{0}}\frac{\left|R_{\eta }-Y\right|}{Y}. \end{array}$$

This difference is due to the assumed inequality |*R*_*η*_ − *Y*| ≪ *Y* not being well-satisfied, as in the case shown in Figure 3D, for example. A sharp peak occurs when *R*_*η*_ ≈ *Y*, and a broader peak is obtained when *Y* decreases.

### 3.4 Mode-dependent gains (*G*-effect)

In previous analyzes, the loop gains *X* and *Y* in Equation 30 have been assumed to be mode-independent (Robinson et al., 2004; Abeysuriya et al., 2014). However, this need not be the case, so long as the following modal version of the relevant stability criterion is satisfied (Robinson et al., 1997; Abeysuriya et al., 2014):


(78) $$\begin{array}{l} X_{\eta }\left(0\right)+Y_{\eta }\left(0\right)<1+k_{\eta }^{2}r_{ee}^{2}. \end{array}$$

These gains can change relatively slowly between states of arousal (Robinson et al., 2004; Abeysuriya et al., 2014, 2015; Assadzadeh et al., 2023), or rapidly due to adaptation during cognitive processes (Babaie-Janvier and Robinson, 2018, 2019, 2020; Robinson et al., 2021a; Babaie-Janvier et al., 2024), the latter case being of most relevance here.

Because *Y*(*ω*) involves the spatially-dependent corticothalamic gains, it varies spatially itself. Hence, we can expand *Y*(*ω*) in terms of eigenmodes and use its expectation value for each mode as we did for *τ*(**r**) in Section 3.2, again neglecting mode-coupling induced by these nonuniform perturbations. Then we find


(79) $$\begin{array}{l} Y\left(\text{r}\right)=\overset{n}{\underset{lm}{\sum }}y_{lm}u_{lm} \end{array}$$

and


(80) $$\begin{array}{l} \langle \eta |Y\left(\text{r}\right)|\eta \rangle =\overset{n}{\underset{lm}{\sum }}y_{lm}J_{lm}^{LM}, \end{array}$$

where the $J_{lm}^{LM}$ are the integrals given in Table 4, and the *y*_*lm*_ are the corresponding expansion coefficients.

Chiang et al. (2008) showed that the EEGs of a significant number of individuals exhibit double alpha peaks that vary in power between the back and front of the head. Replacing *Y*(*ω*) in Equation 32 by the expectation value 〈*η*|*Y*(**r**)|*η*〉, we can then estimate consistent expectation values of *G*_*es*_ and *G*_*se*_ that result in the expected double alpha peak over the head without affecting the stability of the system.

The variation of alpha power over the scalp tends to imply that at least two modes are involved, because the 00 mode is uniform. The main variation is fronto-occipital, with higher power at electrodes of the International 10-20 system (Nunez, 1996; Niedermeyer and Lopes da Silva, 1999) such as O1 than at Cz or Fp1, whose locations are shown in Figure 5A. The approximate coordinates (*ϑ*, *φ*) of these electrodes in our coordinate system are (81°, 109°) for O1, (48°, 327°) for Cz, (164°, 329°) for T3, and (110°, 264°) for Fp1, and in the FreeSurfer coordinate system they become (117°, 82°) for O1, (0°, 180°) for Cz, (112°, 311°) for T3, and (79°, 245°) for Fp1.

Figure 5B shows the individual power spectra of the uniform eigenmode (*η* = 00, blue curve) and second eigenmode (*η* = 1-1, green curve), respectively, where both have default parameters similar to those in previous studies, as listed in Table 1 and the Default row in Table 6. We see clear frequency splitting of 0.9 Hz, due to the *k*-effect discussed in Section 3.1 and consistent with Equation 39, which gives 1.0 Hz for a brain hemisphere with area *A*_*S*_ = 0.07 m^2^ if $k_{1-1}^{2}R_{S}^{2}=1.2$ is used (Robinson et al., 2016). If we integrate over space, as in Equation 28, the individual power spectra must be summed to obtain the total spectrum *P*(*ω*). However, in the Default case, the power in the 00 mode is so dominant that only a single alpha peak results if the two are summed (not shown). This implies that a second alpha peak can only be seen if the gain of the 1-1 mode is increased relative to its default value. Indeed, using the parameters from the Increased Gain case in Table 6, the 1-1 spectrum is enhanced (red curve) to the point that two peaks are seen in the total spectrum (dashed curve). Notably, the new corticothalamic gain parameters *G*_*es*_ and *G*_*se*_ for the 1-1 eigenmode are increased by ~14% and ~35%, respectively, relative to their default values. This can be explained via Equation 71, because a small increase in the corticothalamic loop strength *Y* results in a significant increase in the maximum alpha power (∝ $|R_{\eta }-Y{|}^{-2}$ in the near-critical state when *R*_*η*_ ≈ *Y* and mode *η* is on the verge of instability) and a corresponding decrease in the alpha peak half width (Equation 77), both favoring the appearance of a distinct second alpha peak. Changes of similar magnitude have been inferred to occur between eyes-open and eyes-closed states (Robinson et al., 2002; Abeysuriya et al., 2014), so they are realistic in magnitude. A secondary effect of the enhanced 1-1 gain is that the 1-1 peak frequency increases by ~0.3 Hz relative to the default case.

In Figure 5C we show the spectra *P*(**r**, *ω*) at positions **r** corresponding to the O1, Cz, and Fp1 electrodes on the assumption that the modes are driven in an uncorrelated manner so their individual spectra simply add to give the total. This shows that there is little variation between O1 and Fp1, but a decreased spectrum around Cz, near where the 1-1 mode has a zero, as seen in Figure 2. This topography does not correspond to experiment; as will be seen below, the reason for the discrepancy proves to be the assumption of completely uncorrelated excitation of the modes, which we relax in later sections.

In order to illustrate how single and double alpha peaks depend on parameters other than gains, we next use the parameters from the Small Splitting parameters in Table 6. Specifically, we decrease *r*_*ee*_ to 50 mm, which decreases the frequency separation between the 00 and 1-1 peaks, in accord with Equation 39. Gains are also slightly decreased in this example, with the result that *P*(*ω*) displays only a single peak with a slight shoulder, as seen in Figure 5D. In Figure 5E we see this shoulder in *P*(**r**, *ω*) at front and back of the scalp, but not near Cz, where the 1-1 modal power is small. The shoulder is on the low-frequency side in frontal regions, and the high-frequency side in occipital ones, in line with the relative dominance of lower and upper peaks, respectively. Figures 5F, G are in the same format as 5b and 5c, but with increased *r*_*ee*_ = 70 mm and slightly increased gains (Large Splitting parameters in Table 6). In this case, two sharp peaks separated by 1.1 Hz are clearly seen in *P*(*ω*) and in *P*(**r**, *ω*) at all locations. In Figures 5C, E, G, the 00 peak's contribution to *P*(**r**, *ω*) is close to being spatially uniform, as expected, aside from weak non-uniformities due to varying overlap with the tail of the 1-1 modal contribution. However, the spatial variation of the 1-1 contribution is not consistent with experiment; we resolve this point below.

Overall, we conclude from Figure 5 that (i) the first (*η* = 00, uniform) eigenmode corresponds to the lower frequency alpha peak when there are two peaks, while the second eigenmode (*η* = 1-1) is involved in the higher frequency alpha peak or contributes to the single-peak power if the frequency offset is small; (ii) moderately enhanced 1-1 gain is required for this mode to have a significant contribution; and (iii) the assumption of the excitation of modes being completely uncorrelated is incorrect.

The need for the 1-1 gain to be increased moderately to see a substantial contribution from this mode immediately suggests a mechanism for alpha blocking: a similarly modest reduction in the gain of a near-critical mode can produce a disproportionately large reduction in its power by increasing the size of the resonant denominator in Equation 71 and thus detuning the resonance. This goes equally well for the 00 mode, a point that was previously noted in connection with the blocking of alpha by eye-opening (Robinson et al., 2002). Figure 5 implies that a ~10% decrease in the gains *G*_*es*_ and *G*_*se*_ can result in a ~70% reduction in the alpha power, which is of the correct order to account for the ~80% alpha suppression observed by Hari and Puce (2017), for example.

Based on the definition of posterior alpha blocking that is a suppression in the alpha rhythm that occurs with attention, especially visual stimuli or mental effort (Nunez et al., 1978; Niedermeyer and Lopes da Silva, 1999; Shaw, 2003), we thus argue that these triggers of blocking can be linked to reduction in the gain. This is also consistent with Babaie-Janvier and Robinson (2020), who found that attention to stimuli during evoked responses is associated with corticothalamic gain reductions that tend to reduce alpha, but only considered the 00 mode.

### 3.5 Alpha topography

Having demonstrated that we can model the alpha frequency offsets and blocking observed in EEGs, we now consider the topography of the alpha rhythm in detail. We note that this is the topography of *ϕ*_*ee*_ at the cortical surface, which approximates the source of EEG and MEG. Before reaching the scalp, the resulting fields are distorted by the effects of cortical folding and electric fields are smoothed by volume conduction. However, the effects of volume condition are least for the lowest modes considered here, and do not affect MEG.

It has long been known that the alpha peak is concentrated in occipital regions, but is observable across the whole scalp (Nunez et al., 1978; Niedermeyer and Lopes da Silva, 1999; Shaw, 2003). Based on the work of Chiang et al. (2008), a single alpha peak is observed in a significant number of people, such that its power usually decreases from the back to the front of the head, with a typical ratio of about 1.6:1. However, when split alpha peaks are observed, a number of other key features are seen (Chiang et al., 2011): (i) The frequency of each peak is constant across the scalp; (ii) the sharpness of both peaks can be similar; (iii) the lower frequency peak varies with a typical ratio of about 1.4:1 between the back and front of the head; and (iv) the power in the higher frequency peak is highest in the occipital regions, but falls toward the front with a typical ratio of about 1.8:1 (e.g., see Figure 9 of Chiang et al., 2008).

#### 3.5.1 Alpha power topography via eigenmode expansion

Equations 14, 25 show that the frequency variation of the alpha power is dominated by resonant denominators in the transfer functions *T*(*k*_*η*_, *ω*) of the form $k_{\eta }^{2}+q^{2}\left(\omega \right)$. We can write


(81) $$\begin{array}{l} P\left(\text{r},\omega \right)=\underset{\eta }{\sum }\underset{\mu }{\sum }u_{\eta }\left(\text{r}\right)T\left(k_{\eta },\omega \right)C_{\eta \mu }\left(\omega \right)T^{*}\left(k_{\mu },\omega \right)u_{\mu }\left(\text{r}\right), \end{array}$$

(82) $$\begin{array}{l} C_{\eta \mu }\left(\omega \right)=\langle \phi_{sn}\left(k_{\eta },\omega \right)\phi_{sn}^{*}\left(k_{\mu },\omega \right)\rangle . \end{array}$$

In what follows, we restrict *η* and μ to the first four modes 00, 1-1, 10, and 11, which are the easiest to excite and thus dominate brain activity (Nunez et al., 2001; Robinson et al., 2001, 2016). In the case of excitation of single modes, these spectra correspond to the four curves shown in Figure 3C, where *η* = 00 corresponds to the uniform mode, *η* = 1-1 mainly corresponds to the frontal-to-occipital variation of activity, *η* = 10 predominantly corresponds to the dorsal-to-ventral (top-to-bottom) variation of activity, and *η* = 11 mainly corresponds to the lateral-to-medial (left-to-right in left hemisphere) variation. Note that in Figure 3C all the modes had the same gains as the 00 mode (Table 1), whereas for each case below we use the corresponding new gain parameters for the relevant l=1 mode(s) according to Table 6 (lines 2–8), so the peaks are larger.

#### 3.5.2 Single alpha peak

In the case in which there is a single alpha peak, we now investigate several possibilities for how it is generated. The first such possibility is that only the uniform mode (*η* = 00) is significantly excited, with the others suppressed by the relatively large denominators of their *T*(*k*_*η*_, *ω*) in Equations 70, 71 if *Y*(*ω*) does not depend on *η*. In this case, the power would be spatially uniform, which is contrary to the observation of substantial front-to-back variation of alpha power that is commonly observed when there is a single peak, as in the example in Figure 7B of Chiang et al. (2008), who found a typical power ratio of about 1.6:1. However, the possibility that volume conduction attenuates alpha more strongly in frontal regions cannot be ruled out at this point, an issue we discuss further below.

**Figure 6:**
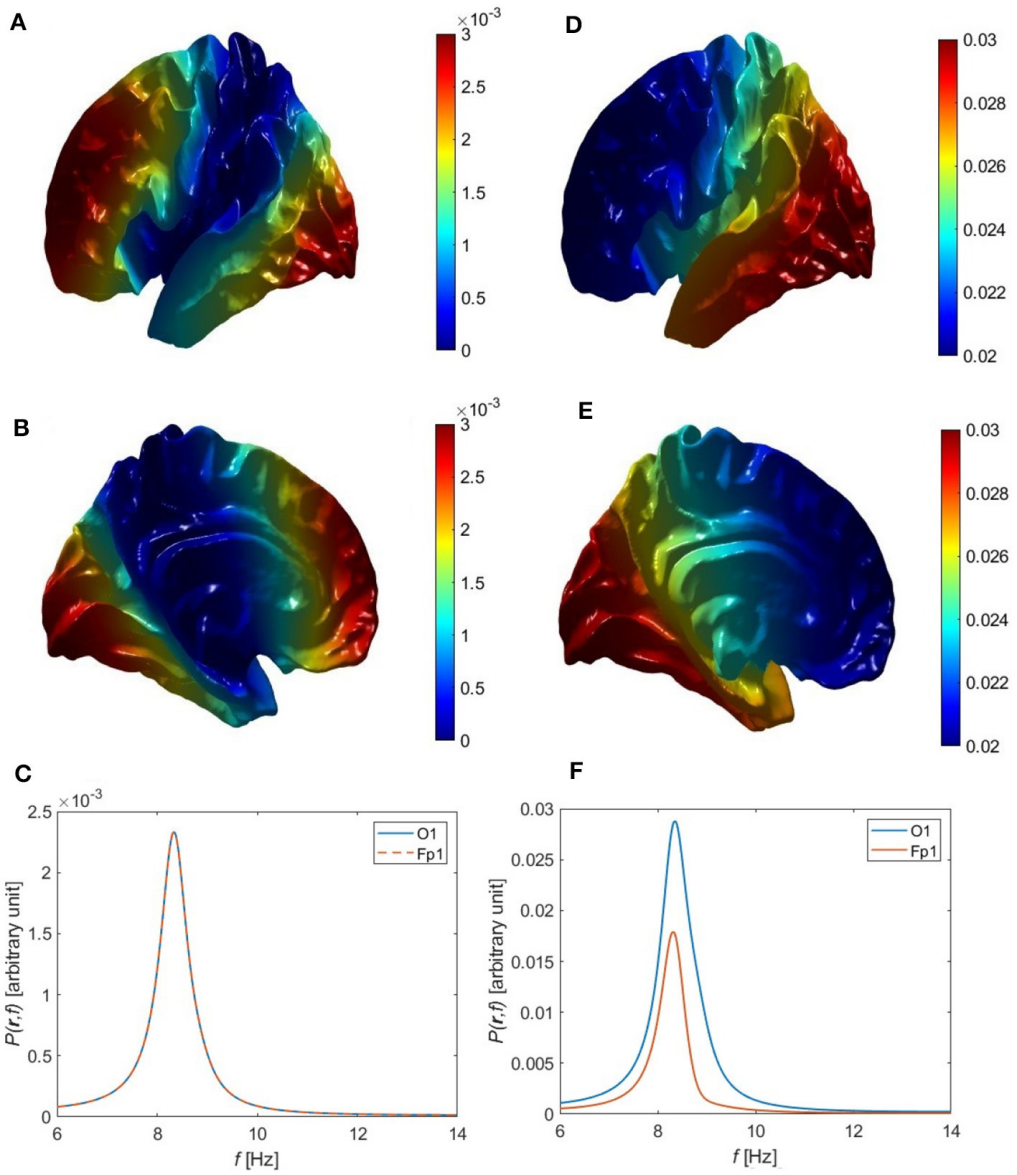
Overall single-peak power topography and spectra for uncorrelated and correlated eigenmodes, using the Single Alpha parameters in Table 6. **(A)** Lateral view of the cortical topography of power at the alpha peak for uncorrelated eigenmodes, from Equation 91. **(B)** Medial view corresponding to **(A)**. **(C)** Power spectra corresponding to **(A)** at the Fp1 (red curve) and O1 (blue curve) electrodes. **(D)** Lateral view of the cortical topography of power at the alpha peak for correlated eigenmodes, from Equation 83. **(E)** Medial view corresponding to **(D)**. **(F)** Power spectra corresponding to **(D)** at the Fp1 (red curve) and O1 (blue curve) electrodes.

The next possibility is that some or all of the next three non-uniform modes are also excited while overlapping in frequency, so that only a single peak is seen. If the excitation of these modes were random-phase, the resulting power distribution would be almost symmetric between front and back of the head, as seen in Figures 6A–C for the Single Alpha parameters in Table 6, because the power spectrum then depends on the squares of the eigenmodes via Equation 70. Thus, uncorrelated excitation cannot account for the observed concentration of alpha in the occipital regions.

For the alpha power to be strongest in occipital regions, at least one of the first three non-uniform modes together with the uniform eigenmode must be excited such that they are in phase at the back of the head and out of phase at the front. This is consistent with the fact that variations in gains and other quantities break any residual fronto-occipital symmetry and favor modes whose excitations have the same sign in the relevant sensory region, in this case V1. (This is analogous to the situation when a violin string is plucked: modes are excited with the same sign at the point of plucking.) We analytically approximate this in-phase dynamics of the eigenmodes by setting *T*(*k*_*η*_, *ω*) = *a*_*η*_*T*(0, *ω*) in the vicinity of their common peak, which approximates the spectral shape of each transfer function as being the same, with *a*_*η*_ allowing for their amplitudes relative to the uniform mode. Hence, *C*_*η*ν_ = *a*_μ_*a*_*η*_ in Equation 81 for these modes and Equation 81 yields


(83) $$\begin{array}{l} P\left(\text{r},\omega \right)\approx |T\left(0,\omega \right){|}^{2}{\left[u_{00}\left(\text{r}\right)+a_{1-1}u_{1-1}\left(\text{r}\right)+a_{10}u_{10}\left(\text{r}\right)+a_{11}u_{11}\left(\text{r}\right)\right]}^{2}. \end{array}$$

We can obtain useful insight into Equation 83 by considering the idealized case of a truly spherical cortex such that the modes align spatially as closely as possible with those of the convoluted cortex, as discussed in Section 3.2. In this case, the spherical harmonics are the eigenmodes, and Equation 83 can be simplified to


(84) $$\begin{array}{c} P\left(\text{r},\omega \right)\approx \frac{|T\left(0,\omega \right){|}^{2}}{4\pi }\\ {\left[1+a_{1-1}\sqrt{3}\sin\vartheta \sin\varphi +a_{10}\sqrt{3}\cos\vartheta +a_{11}\sqrt{3}\sin\vartheta \cos\varphi \right]}^{2}; \end{array}$$

this form provides valuable guidance in the convoluted-cortex case because of the similarity of the eigenmodes discussed in Section 3.2. In obtaining Equation 84 we have used


(85) $$\begin{array}{l} u_{00}=\sqrt{\frac{1}{4\pi }}, \end{array}$$

(86) $$\begin{array}{l} u_{1-1}=\sqrt{\frac{3}{4\pi }}\sin\vartheta \sin\varphi , \end{array}$$

(87) $$\begin{array}{l} u_{10}=\sqrt{\frac{3}{4\pi }}\cos\vartheta , \end{array}$$

(88) $$\begin{array}{l} u_{11}=\sqrt{\frac{3}{4\pi }}\sin\vartheta \cos\varphi . \end{array}$$

The modes most strongly driven by visual stimuli are 00 and 1-1 because neither has a zero in or near V1. In this case, *a*_10_ = *a*_11_ = 0 in Equations 83, 84, as noted in Table 6, and the observed ratio of power at frontal and occipital electrodes can be used to estimate *a*_1 − 1_. For a convoluted cortex, the power ratio can be expressed by


(89) $$\frac{P(r_{\text{O1}},\omega )}{P(r_{\text{Fp1}},\omega )}\approx {\left[\frac{u_{00}(r_{\text{O1}})+a_{1-1}u_{1-1}(r_{\text{O1}})}{u_{00}(r_{\text{Fp1}})+a_{1-1}u_{1-1}(r_{\text{Fp1}})}\right]}^{2};$$

for a spherical cortex, this result simplifies to


(90) P(rO1,ω)P(rFp1,ω)≈[1+a1 − 13 sinϑO1sinφO11+a1 − 13 sinϑFp1sinφFp1]2.

Thus, the power ratio depends on the locations of the electrodes and the amplitudes of the eigenmodes at those points.

Upon substituting a typical power ratio of 1.6:1 into Equation 90, using the electrode coordinates listed in Section 3.4, and solving for *a*_1 − 1_, we find *a*_1 − 1_ = − 0.08 in this case, which is small compared to the unit amplitude of the 00 mode. The negative sign of *a*_1 − 1_ is expected because Figure 2 shows that the 1-1 eigenmode has a front to back variation with the positive region at the front, but this is only determined to within an overall sign change. Hence, if superposed with the first eigenmode in Equation 83 without a sign reversal, the resultant alpha power would be stronger in front, which is contrary to experiment. Thus, the negative sign in *a*_1 − 1_ reverses the polarity of the 1-1 eigenmode resulting in the expected concentration of power in occipital regions as it places the modes in-phase at the back of the head and out of phase at the front when driven together by the same external inputs. Figures 6D–F demonstrate that this combination of the first two eigenmodes (00 and 1-1) for a convoluted cortical surface yields power spectra with a single alpha peak such that the alpha power is higher at O1 than Fp1, with a ratio consistent with the experimental power spectra in Figure 7B of Chiang et al. (2008). The corresponding cortical topography of the alpha power is shown in Figures 6D, E, which clearly show the decrease of the alpha power from the back to the front of the head. The O1 and Fp1 spectra in Figure 6F show that the peak power differs by a factor of ~1.6 in this example, consistent with typical values from experiment.

#### 3.5.3 Two alpha peaks

There are several possibilities for how two alpha peaks might be generated, but in all cases the uniform 00 mode must be excited to account for the low frequency peak. In addition to this, the first possibility is that one of the *l* = 1 modes (i.e., a mode with *η* = *lm* and *l* = 1) is significantly excited, resulting in a second peak with higher alpha frequency (based chiefly on the *k*-effect as discussed earlier in Section 3.1). A third possibility is that two or all of the *l* = 1 modes, overlapping in frequency, are excited to produce the higher frequency peak.

To see two distinct alpha peaks, the widths of their peaks must be less than about half their separation. Hence, for peaks that are non-overlapping in frequency, if *ω*_00_ < *ω*_1 − 1_ ≈ *ω*_10_ ≈ *ω*_11_, then


(91) $$\begin{array}{c} P\left(\text{r},\omega \right)\approx |T\left(0,\omega \right){|}^{2}\\ {\left[u_{0}\left(\text{r}\right)\right]}^{2}+|T\left(k_{1-1},\omega \right){|}^{2}{\left[a_{1-1}u_{1-1}\left(\text{r}\right)+a_{10}u_{10}\left(\text{r}\right)+a_{11}u_{11}\left(\text{r}\right)\right]}^{2}, \end{array}$$

where we have approximated the frequency dependence of the transfer functions of the second to fourth modes as being the same because of the small frequency differences between *l* = 1 modes obtained from Equation 39; amplitude differences are absorbed into the *a*_*lm*_. In essence, the non-overlapping 00 and *l* = 1 peaks can be treated as being mutually uncorrelated, so long as the observation time is long enough to distinguish their frequencies. In the spherical approximation, Equation 91 simplifies to


(92) $$\begin{array}{c} P\left(\text{r},\omega \right)\approx \frac{|T\left(0,\omega \right){|}^{2}}{4\pi }+\frac{3|T\left(k_{1-1},\omega \right){|}^{2}}{4\pi }\\ {\left[a_{1-1}\sin\vartheta \sin\varphi +a_{10}\cos\vartheta +a_{11}\sin\vartheta \cos\varphi \right]}^{2}. \end{array}$$

We start with the simplest case in which only the first two modes are excited, and we set *a*_10_ = *a*_11_ = 0 in Equations 87, 88. Increasing the gains for the 1-1 eigenmode to the values for the Double Alpha case in Table 6 yields a power spectrum with two alpha peaks as in Section 3.6.3. However, the upper peak's alpha power at points O1 and Fp1 is roughly the same in this case, so the combination of just the first two eigenmodes is not sufficient to reproduce the observed front-to-back variation of the alpha power in the upper peak.

**Figure 7:**
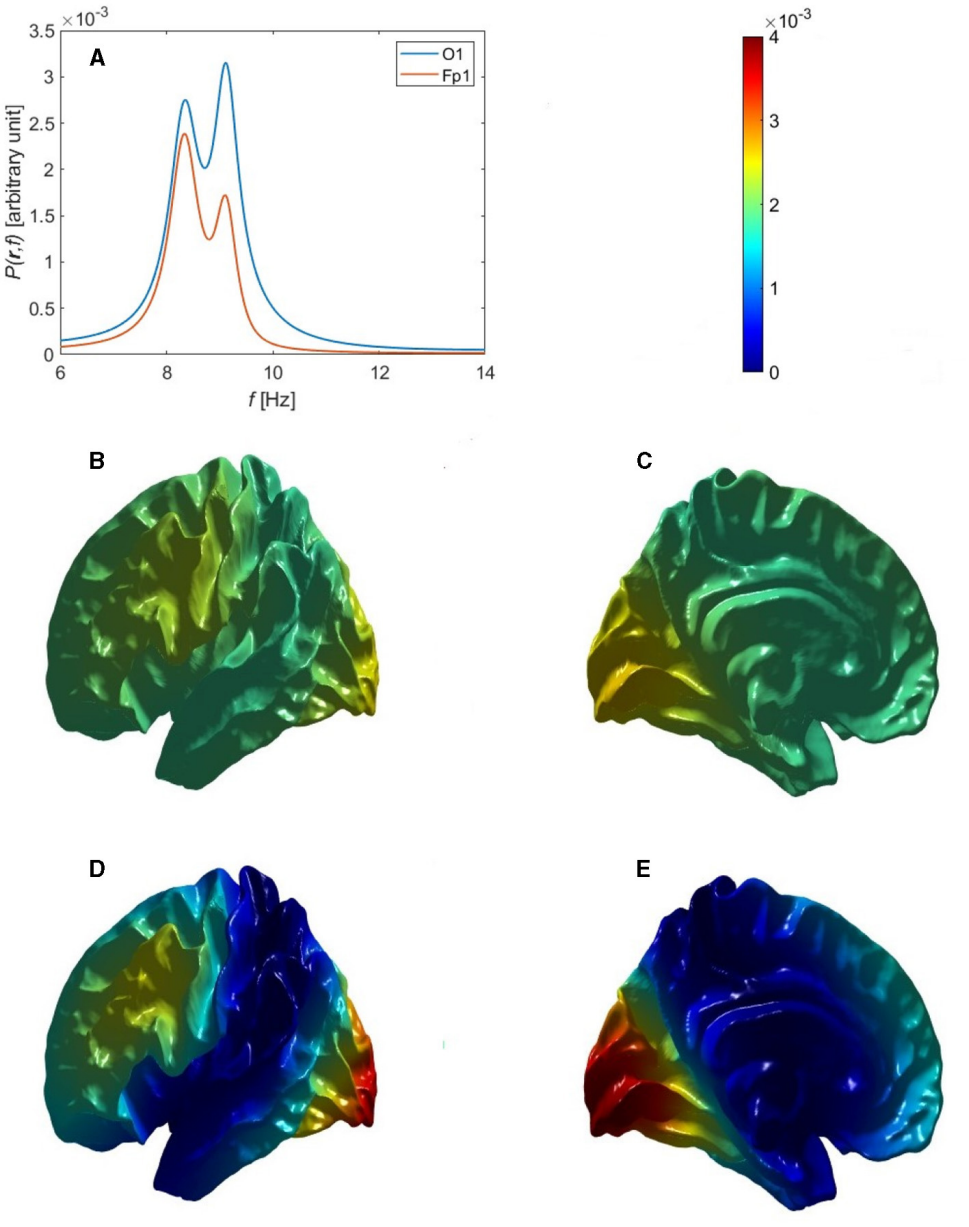
Overall power spectrum and alpha topography when exciting the 00, 1-1, and 11 modes with the Double Alpha parameters in Table 6. **(A)** Overall power spectrum at O1 and Fp1 electrodes, from Equation 91. **(B)** Lower-frequency peak's cortical topography, lateral view. **(C)** Medial view corresponding to **(B)**. **(D)** Higher-frequency peak's cortical topography, lateral view. **(E)** Medial view corresponding to **(D)**.

Next, by analogy with the mechanism that produced fronto-occipital variation in the power for a single alpha peak, we consider the case when two modes contribute to the upper alpha peak. Figure 5A shows that V1 does not lie on the nodal line of the 11 mode, so we add a contribution from the 11 mode using the Double Alpha parameters in Table 6. Figure 7A shows that the resulting power spectrum has two peaks with frequencies of around 8.4 and 9.1 Hz. The upper peak has a power ratio of about 1.85:1 between the O1 and Fp1 electrodes, which reproduces the typical value in Figure 9 of Chiang et al. (2008). Figures 7B, C show that the lower-frequency peak also has a front-to-back variation of alpha power but with a smaller ratio of about 1.15:1, which less than the typical 1.4:1 ratio from Figure 7C of Chiang et al. (2008) and is reflected in the relatively uniform topography seen in Figures 7B, C here; however, we note that our parameters can be adjusted to give somewhat larger or smaller non-uniformities, so it is only the order of magnitude of the deviation from uniformity that is at issue here. There is a weak spatial variation that arises from the tail of the upper peak having a small and variable overlap with the lower peak, leading to a slight occipital power enhancement.

The higher experimental front-to-back variation of power seen by Chiang et al. (2008) at the lower alpha peak may be due to the variation of the skull and scalp thicknesses between the back and the front of the head (Mahinda and Murty, 2009; Thulung et al., 2019), which in turn affect the measured EEG signal because of attenuation by volume conduction; alternatively, neurons may be less efficient at generating EEG signals at the front of the head because of differences in size or alignment (Nunez and Srinivasan, 2006). If the lower peak is assumed to be due to the 00 mode, its power variation provides a measure of these combined effects. This would imply that the power variation at the cortex would be only 1.15/1.4 ≈ 0.82 as large as that at the scalp. So typical single-peak power variations in the previous section would be reduced from 1.6 to around 1.3 at the cortex and the upper-peak variation would be only around 1.5 rather than 1.85. This would reduce the required amplitudes *a*_1*m*_ of the *l* = 1 modes accordingly, but we do not recalculate these values here because such refinements would add complexity without affecting the main conclusions.

The power topography at the upper alpha peak is shown in Figures 7D, E, which demonstrate strong occipital localization of the power, with a slight frontal enhancement and very little power near the temporal and central electrodes, which lie outside V1 (dark blue area). The latter feature was not seen in the example shown in Figure 7 of Chiang et al. (2008), which implies some involvement of the 10 mode, which peaks in that region.

### 3.6 Mu rhythm

**Figure 8:**
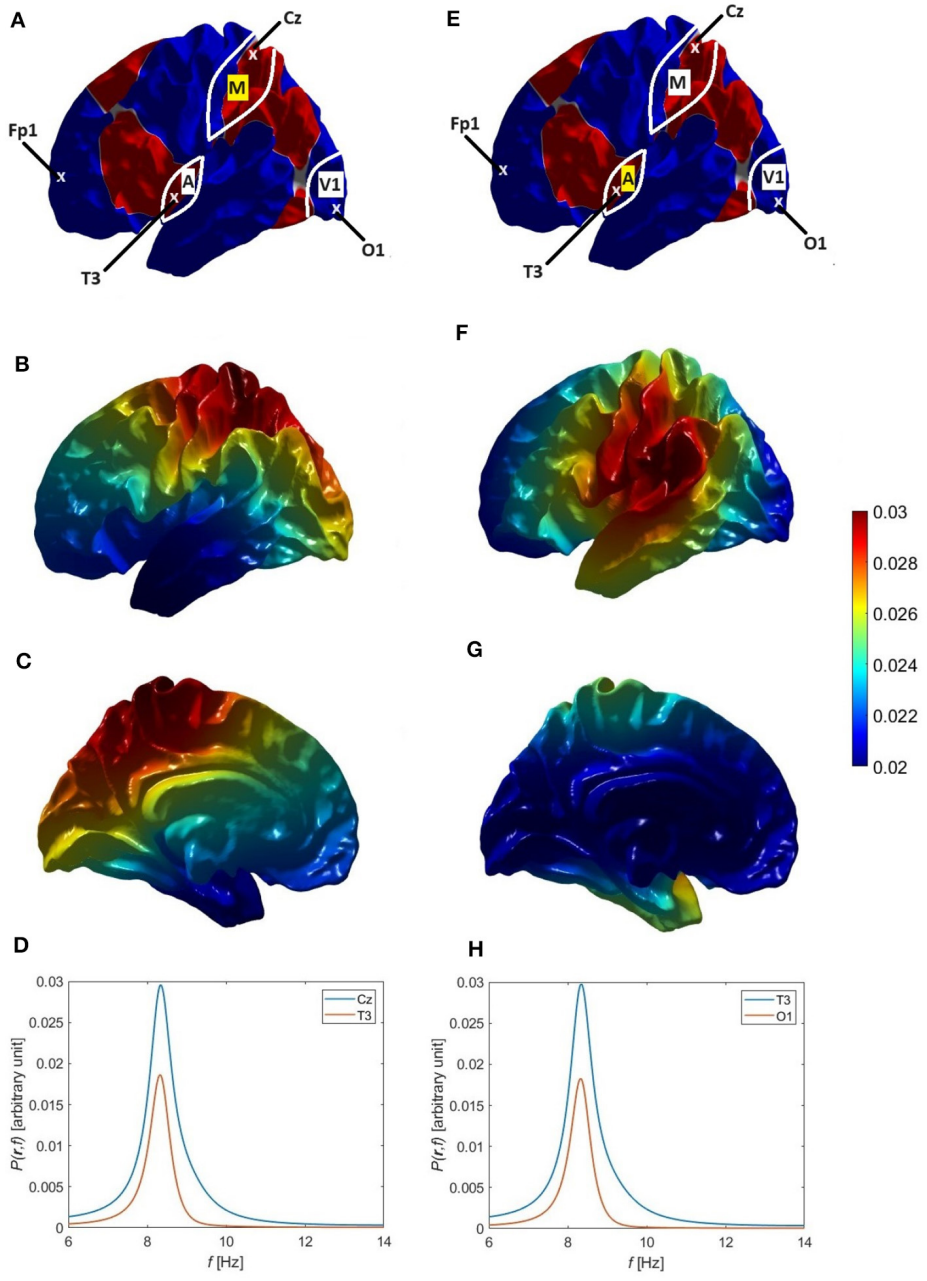
Mu and tau rhythm topography and power spectra obtained for the Mu and Tau parameters, respectively, in Table 6. **(A)** Left hemisphere lateral view, showing key electrode locations, with sensorimotor cortex highlighted. **(B)** Mu cortical power topography, lateral view. **(C)** Medial view corresponding to **(B)**. **(D)** Mu power spectra at Cz (red curve) and T3 (blue curve) sites. **(E)** Left hemisphere lateral view, showing key electrode locations, with auditory cortex highlighted. **(F)** Tau cortical power topography, lateral view. **(G)** Medial view corresponding to **(F)**. **(H)** Tau power spectra at O1 (red curve) and T3 (blue curve) sites.

The rolandic (or central) mu rhythm is a rhythm that lies within the alpha band. The mu rhythm is classically defined as an 8–12 Hz rhythm that is strongest over the central sensorimotor regions of the head, labeled M in Figure 8A, and is suppressed or blocked by movement (Gastaut et al., 1952; Pfurtscheller and Aranibar, 1979; McFarland et al., 2000; Garakh et al., 2020).

The single-peak alpha rhythm results in Section 3.5.2 showed that the uniform eigenmode together with a mode that has a front-to-back variation are sufficient to reproduce the corresponding occipito-frontal alpha topography if the two modes are in phase in the relevant sensory region. Hence, we use an analogous eigenmode superposition to reproduce the concentration of mu power in central regions near the sensorimotor cortex.

If the uniform eigenmode (*η* = 00) is excited together with an *l* = 1 eigenmode, the latter must have *η* = 10 in order to account for the variation in mu power because this mode has primarily a dorso-ventral variation, as shown in Figure 2F. Hence, setting *a*_1 − 1_ = *a*_11_ = 0 in Equation 83 with *a*_10_ = − 0.09, as for the Mu parameters in Table 6, yields the topography shown in Figures 8B, C in lateral and medial views, respectively. These figures show a dorso-ventral variation that reproduces the experimentally observed concentration of mu power in regions near the sensorimotor cortex (Garakh et al., 2020). Note that the negative sign in *a*_10_ reverses the polarity of the 10 eigenmode, resulting in the expected concentration of power in dorsal regions because it places the modes in-phase at the top of the head and out of phase at the bottom when driven together in the somatosensory region, adjacent to the primary motor cortex. Figure 8D shows a power ratio of circa 1.6:1 between the Cz and T3 electrodes. Reduction of modal gains *G*_*es*_ and *G*_*se*_ by ~10% results in a ~70% reduction in mu power near Cz, consistent with the ~60% mu suppression observed in the experiment by Van Leeuwen et al. (1978).

### 3.7 Tau rhythm

Another rhythm within the alpha band is the tau rhythm, which is typically an 8–10 Hz rhythm detected by EEG and MEG over the temporal regions of the head, and which is suppressed or blocked by auditory stimuli, which are received at the auditory cortex, marked A in Figure 8E (Tiihonen et al., 1991; Yokosawa et al., 2020). As in previous sections, we can superpose two eigenmodes to reproduce the concentration of tau power in temporal regions near the auditory cortex.

We again argue that the uniform eigenmode (*η* = 00) is excited together with an *l* = 1 eigenmode. Knowing that the *l* = 1 eigenmode should contribute to the medio-lateral variation in tau power implies that we should choose this mode to be *η* = 11, which has a predominantly left-to-right variation as shown in Figure 2G. Setting *a*_1 − 1_ = *a*_10_ = 0 in Equation 83 with *a*_11_ = 0.32, as for the Tau parameters in Table 6, yields the topography shown in Figures 8F, G in lateral and medial views, respectively. These figures show a medio-lateral variation that reproduces the observed concentration of tau power in temporal regions near the auditory cortex and the T3 electrode, consistent with experiment (Tiihonen et al., 1991; Yokosawa et al., 2020).

Tau blocking can be reproduced by decreasing the gain of the corresponding eigenmodes, as in the analyzes in earlier sections. The power spectra formed by the superposition of the 00 and 11 modes for the gain parameters in Table 1 for both eigenmodes and for the case of a 10% drop in their corresponding gain parameters (*G*_*es*_ and *G*_*se*_) by ~10% results in ~70% suppression of the tau power near temporal regions, consistent with experiments by Tiihonen et al. (1991), who found an 80% reduction.

## 4 Summary and discussion

A century after the first human EEG observations and discovery of the alpha rhythm, we have used just the first four cortical eigenmodes to formulate the first compact unified description of how the alpha, mu, and tau rhythms of healthy awake individuals are generated and distributed over the cortical surface. Eigenmode analysis provides a parsimonious explanation of the spectral structure of these rhythms, including peak splitting, cortical power topography, and the relationships to underlying physiology, including a plausible mechanism for blocking. Our main results are as follows:

(i) The alpha power variation over the scalp and the alpha frequency offset observed in EEGs with a split alpha peak are found to be consequences of three effects:

- Each eigenmode has a different eigenvalue and characteristic wave number *k*. Hence, its corresponding alpha frequency peak is shifted relative to that of the uniform 00 mode, whose frequency arises from time delays in corticothalamic loops, as in prior work (Robinson et al., 2002, 2016). The relative shifts in the alpha frequencies of other modes are approximated in terms of the physiological parameters of NFT and found to be ~1 Hz, which is consistent with numerical NFT values, and agrees with the observed ~1–2 Hz alpha frequency offset in subjects characterized by a double alpha peak (Nunez, 1996; Niedermeyer and Lopes da Silva, 1999; Nunez et al., 2001; Chiang et al., 2008, 2011); hence, there is a secondary role for cortical modes in determining alpha frequencies, rather than the primary one originally suggested by Nunez et al. (1978). Large frequency splittings are predicted to be favored for small brains with relatively long and fast corticocortical white matter axons. Notably, the fact that all brains should exhibit higher frequencies for *l* = 1 modes than for the 00 mode is consistent with the observations by Chiang et al. (2008) and Chiang et al. (2011) that such splittings are ubiquitous, but sometimes too small to be resolved in short EEG recordings.
- Non-uniformities in the corticothalamic loop delay (*τ*) that were incorporated via the expectation value of *τ* for each spatial eigenmode by treating the dependence of the eigenmodes on *τ* as a perturbation from its mean value. However, because of partial cancelation of positive and negative contributions, this effect is found to be too small to account for the alpha frequency shift unless the perturbation is unrealistically large, thus arguing against this being the primary mechanism for alpha splitting, contrary to the proposal by Robinson et al. (2003) and Chiang et al. (2008, 2011).
- Introducing mode dependence to physiological gains, we found that higher corticothalamic gains for *l* = 1 modes (here the second and fourth eigenmodes *η* = 1-1 and *η* = 11 were considered) is essential for the appearance of a distinct second alpha peak in the overall power with a topography consistent with experiment [see also point (iii) below].

(ii) The necessity of increasing the gain of the higher eigenmodes to obtain a second distinct alpha peak suggested a potential mechanism of alpha blocking, in which reducing the corticothalamic gain of modes results in the suppression of the associated alpha peak. Because the brain is close to criticality when the alpha peak is strong, even a ~10% reduction in corticothalamic loop gains suffices to reduce the alpha power by ~70% in the cases considered. Such a reduction is also consistent with the work of Babaie-Janvier and Robinson (2020), who found that rapid reductions in corticothalamic gain occur in response to stimuli in evoked-response experiments but who considered only the 00 mode.

(iii) We found that just the 00, 1-1, and 11 eigenmodes suffice to reproduce the observed topography of the classical alpha rhythm in both cases of subjects exhibiting a single alpha peak or a double alpha peak, such that:

- For the single alpha peak case, the 00 and 1-1 eigenmodes, overlapping in frequency, were found to be necessary and sufficient to reproduce the observed alpha topography between the front and back of the head with a fronto-occipital ratio consistent with the one obtained in previous experiment by Chiang et al. (2008). These are the first two eigenmodes, where the first (*η* = 00) eigenmode is uniform across the cortex and the second eigenmode (*η* = 1-1) contributes to the front-to-back variation through interference with the 00 mode. We also found that these modes are excited in-phase in the visual sensory region at the back of the head, so they are out of phase at the front; this phase relationship is required in order to obtain the alpha topography observed in experiments. Significantly, the mode amplitudes sum, not their powers, and modal interference is essential to reproducing the observed topography. This removes any need for there to be a localized “generator” or “pacemaker” for the alpha (or mu or tau) rhythm—delocalized modes interfere constructively in occipital regions, and destructively in frontal regions, to produce the observed topography.
- For the double alpha peak case, three eigenmodes were found to be necessary and sufficient to reproduce the observed topography in experiments (Chiang et al., 2008). The required eigenmodes are the first (00), second (1-1), and the fourth (11), with the latter two overlapping in frequency at a higher frequency than the 00 mode; a contribution from the 10 mode is also possible. Since the first eigenmode has a lower frequency than the 1-1 and 11 eigenmodes (due to the *k*-effect), we have argued that it contributes to the lower alpha frequency peak, which is approximately uniform across the cortex; whereas, the other two eigenmodes contribute to the upper alpha peak. A third eigenmode was essential to reproduce the front-to-back variation of the upper peak after the superposition of modes, because the 1-1 eigenmode alone yields a power spectrum that is nearly symmetric between the back and front of the head. Again, the observed topography is accounted for by modal superposition.

(iv) In the case of split-alpha spectra, the lower peak provides a way of estimating front-to-back differences in the ratio of EEG signal to mode amplitude due to effects such as volume conduction or differences in the efficiency of generation. Initial results imply that a given cortical activity level produces a scalp signal about 15% weaker at the front of the head than at the back, which could be used to refine estimates of the amplitudes of the *l* = 1 modes required to obtain the observed alpha topography. However, overlap with the tails of the *l* = 1 modes means that detailed numerical fitting will be necessary to analyze such effects.

(v) We found that superposition of two eigenmodes overlapping in frequency is sufficient to reproduce the observed topography of the rolandic mu rhythm; these are the first (*η* = 00) eigenmode and the third (*η* = 10) eigenmode that has a top-to-bottom variation. Accordingly, we inferred that the 10 mode contributes to the dorso-ventral variation such that the mu power is concentrated in regions near the Cz electrode, and falls toward ventral regions, which is consistent with observations (Gastaut et al., 1952; Pfurtscheller and Aranibar, 1979; Garakh et al., 2020). We also applied our proposed mechanism of blocking on mu rhythm by reducing the gain of the two involved eigenmodes, and reproduced the typical observed suppression of the mu peak, with only a ~10% reduction of corticothalamic gain.

(vi) Two eigenmodes overlapping in frequency also suffice to reproduce the observed topography of the tau rhythm (Tiihonen et al., 1991; Yokosawa et al., 2020). These are the first (00) eigenmode and the fourth (11) eigenmode that has a left-to-right variation. Accordingly, we inferred that superposition of the 00 and 11 modes can account for the tau power being concentrated in temporal regions, which is consistent with what is observed experimentally. We also applied our proposed mechanism of blocking on tau rhythm by reducing the gains of the two involved eigenmodes, and reproduced the observed ~70% suppression of the tau peak, via ~10% corticothalamic gain reductions.

(vii) The above points imply that the existence of three distinct alpha-band rhythms is closely linked to the existence of exactly three members of the lowest (*l* = 1) family of nonuniform eigenmodes. Most importantly, in the single-peak case, superposition of each one of these modes with the 00 mode gives rise to the localized enhancement of activity near V1 (alpha, 1-1 mode); sensorimotor cortex (mu, 10 mode), and auditory cortex (tau, 11 mode).

(viii) A spherical approximation of the cortical surface provides a useful guide when evaluating surface integrals of eigenmodes used to calculate the expectation values for the corticothalamic loop delays for each eigenmode, and when finding the amplitudes coefficients necessary for investigating the alpha topography over the cortex; however, exact symmetries of the sphere are broken in the convoluted cortex. By approximating the NFT predictions for power near each modal alpha frequency, we also derived expressions for the width of the alpha peak at half maximum for each mode and dependence of peak power on gain.

Overall, this paper provides a compact, unified analysis of the frequencies and topographies of human brain alpha, mu, and tau rhythms using just the first four eigenmodes of brain activity. It underlines the value of modal analysis via neural field theory and of the importance of eigenvalue effects, interference between superposed modes, and proximity to criticality. Moreover, its success reinforces arguments against the use of a new *ad hoc* “pacemaker” or “generator” for each enhancement of alpha in frequency and/or space (Nunez and Srinivasan, 2006; van Albada and Robinson, 2013); nor is there any need for general inhibition of the rest of the cortex by regions in which alpha is strong, contrary to early suggestions (Berger, 1929; Gloor, 1969). The results support the role of corticothalamic loops in producing alpha, mu, and tau rhythms, and the role of the cortex in determining the splitting of spectral peaks.

The present work could be extended by exploring the mu and tau rhythms in more detail; for instance, we have found no reports of split mu and/or tau peaks, whereas our results imply that increasing the gain of the relevant *l* = 1 mode can result in a second distinct peak. Hence, we predict the possibility of split mu and/or tau peaks that might be detected in the EEGs of a sufficiently large set of subjects. Another extension could be a deeper analysis of EEGs on an individual level by fitting the present theory to spectra and topography to estimate relative amplitudes of modes, and hence constrain parameter values and their changes during blocking. Similarly, individual modal amplitudes could be projected out, using the analog of Equation 26 with *ϕ*_*ee*_ in place of *ϕ*_*sn*_, to assist in identifying the presence of particular rhythms. Also, by considering differing effects of volume conduction and skull thickness between the back and front of the head we could refine our predictions of the alpha power ratios. Another future avenue could be to relate alpha blocking to attention using eigenmodes by linking our results more closely to those of Babaie-Janvier and Robinson (2020) on the dynamic downregulation of corticothalamic alpha gains associated with attention and by extending their results to multiple modes.

Other directions of interest are to explore the relative topographies of alpha- and beta-band rhythms under conditions of both spontaneous activity and blocking due to stimuli or imagery [so-called event-related desynchronization and synchronization effects (Niedermeyer and Lopes da Silva, 1999)], as well as generalizing recent control-theory analyzes of the 00 resonances and their contributions to evoked responses (Babaie-Janvier and Robinson, 2018, 2019, 2020) to examine the simultaneous dynamics of multiple modal contributions, their interactions, and their effects on prediction and attention.

## Data Availability

Publicly available datasets were analyzed in this study. These data are found in the original references cited.
